# Dynamic Axonal Translation in Developing and Mature Visual Circuits

**DOI:** 10.1016/j.cell.2016.05.029

**Published:** 2016-06-30

**Authors:** Toshiaki Shigeoka, Hosung Jung, Jane Jung, Benita Turner-Bridger, Jiyeon Ohk, Julie Qiaojin Lin, Paul S. Amieux, Christine E. Holt

**Affiliations:** 1 Department of Physiology, Development and Neuroscience, University of Cambridge, Downing Street, Cambridge CB2 3DY, UK; 2 Department of Anatomy, Brain Research Institute, and Brain Korea 21 PLUS Project for Medical Science, Yonsei University College of Medicine, Seoul 03722, Republic of Korea; 3 Bastyr University Research Institute, Bastyr University, Kenmore, WA 98028, USA

## Abstract

Local mRNA translation mediates the adaptive responses of axons to extrinsic signals, but direct evidence that it occurs in mammalian CNS axons in vivo is scant. We developed an axon-TRAP-RiboTag approach in mouse that allows deep-sequencing analysis of ribosome-bound mRNAs in the retinal ganglion cell axons of the developing and adult retinotectal projection in vivo. The embryonic-to-postnatal axonal translatome comprises an evolving subset of enriched genes with axon-specific roles, suggesting distinct steps in axon wiring, such as elongation, pruning, and synaptogenesis. Adult axons, remarkably, have a complex translatome with strong links to axon survival, neurotransmission, and neurodegenerative disease. Translationally co-regulated mRNA subsets share common upstream regulators, and sequence elements generated by alternative splicing promote axonal mRNA translation. Our results indicate that intricate regulation of compartment-specific mRNA translation in mammalian CNS axons supports the formation and maintenance of neural circuits in vivo.

## Highlights

• Dynamic translatome of retinal axons in vivo matches changing subcellular function
• Adult CNS axons translate mRNAs for synaptic transmission and axon survival in vivo
• Target mRNAs of key translation regulators show developmental co-regulation
• Axon-specific sequence motifs link alternative splicing to axonal translation

## Graphical Abstract

**Figure:**
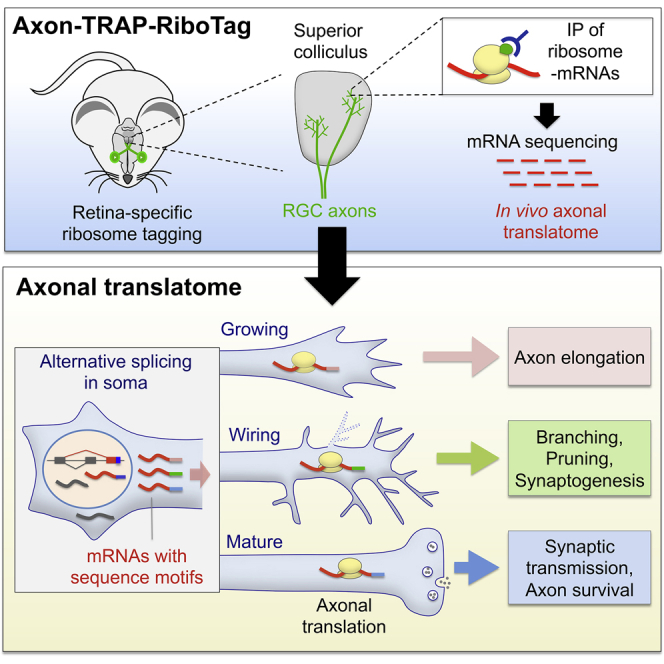

## Teaser

Local mRNA translation in axons of developing and adult CNS neurons in vivo shows dynamic regulation, suggesting functional relevance for neural circuit formation and maintenance.

## Introduction

RNA localization and local translation are evolutionarily conserved mechanisms employed by cells to control the precise subcellular positioning of nascent proteins. Neurons are highly compartmentalized cells with functionally distinct cytoplasmic/membrane domains (dendrites, axons, and somas), and emerging evidence indicates that localized mRNA translation supports this subcellular differentiation (Holt and Schuman, 2013, Martin and Ephrussi, 2009). Recent in vitro studies revealed an unexpectedly large population of mRNAs in axons, and inhibiting the translation of just one or two of them can cause specific defects in fundamental axonal behaviors, such as neurotrophin-induced outgrowth, branching, cue-induced chemotropic responses, and injury-induced regeneration (references in Jung et al., 2012). In vitro studies have also provided evidence that extrinsic signals, such as guidance cues and growth factors, selectively induce rapid axonal synthesis of distinct protein subsets (references in Jung et al., 2012). A rational interpretation of these results is that specific subsets of mRNAs are coordinately translated when required whereas most axonally localized mRNAs remain translationally repressed. Thus, to understand the function of axonal mRNA translation, it is important to carry out a comprehensive and unbiased global analysis of the mRNAs that are specifically translated in the axonal compartment in vivo.

**Figure 1:**
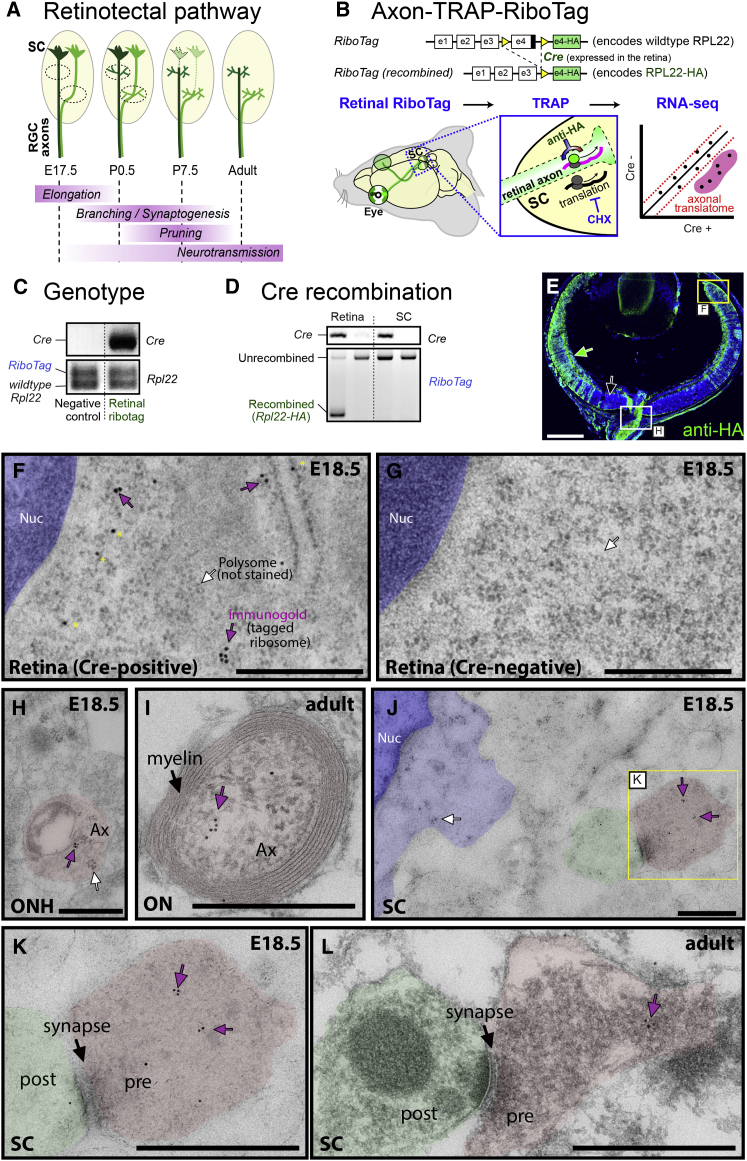
Retinal RiboTag Labels RGC Axonal Ribosomes In Vivo (A) Development of retinal ganglion cell (RGC) axons in the superior colliculus (SC). (B) Strategy of axon-TRAP. (C) PCR detection of *Cre* transgene (upper) and *Rpl22* allele (lower). (D) PCR of genomic DNA from the retina and the SC that distinguish recombined and unrecombined *RiboTag* alleles. (E) HA fluorescence immunohistochemistry. (F–L) HA immuno-gold electron microscopy (EM). HA-tagged ribosomes localize to retinal cell bodies (F) and RGC axons (Ax) in the optic nerve head (ONH) (H), optic nerve (ON) (I), and RGC axon terminals in the SC (J and K). Two or more adjacent gold particles (purple arrows) were regarded as specific signals. Scattered single immuno-gold particles may be non-specific (yellow asterisks). Ultrastructure of polysomes is visible in the cell bodies in the retina and the SC (white arrows), but these co-localize with immuno-gold only in the retina (F). Cre-negative littermate shows no specific labeling (G). E, embryonic day; Nuc, nucleus; P, postnatal day. The scale bars represent 500 μm (E) and 500 nm (F–L). See also Figure S1.

The axons of retinal ganglion cells (RGCs) terminate in the superior colliculus (SC) of the midbrain. A point-to-point topographic projection of RGC axons to the SC allows the brain to reconstruct a map of the outside world. In mouse, the formation of this retinotopic map in the SC can be divided into three distinct phases (Feldheim and O’Leary, 2010). First, embryonic RGC axons enter the SC and initially extend beyond their topographically correct “termination zones (TZs)” without branching or synapsing (“elongation” period). Second, interstitial branches arise from the primary axon shafts of RGCs in their appropriate TZs and begin to form synapses (“branching/synaptogenesis” period). Third, in the first 2 postnatal weeks, correctly wired axon branches are strengthened and excess inappropriate branches are pruned (“pruning” period), resulting in the mature topographic map in adulthood (Figure 1A; Godement et al., 1984). Intriguingly, evidence suggests that local mRNA translation in the RGC axons may regulate subtle aspects of the formation of the retinotectal projection in vivo (Brunet et al., 2005). It is not known, however, which mRNAs are axonally translated and which specific aspects of visual circuit assembly they affect.

To address this issue, we developed axon-TRAP (translating ribosome affinity purification) in mouse, a method that allows specific isolation of ribosome-bound mRNAs in the distal compartment of RGC axons in vivo. Analysis of these axon-specific translatomes at multiple ages reveals that axonal translation may play two major roles: regulation of protein and energy homeostasis, which is supported by mRNAs constitutively translated regardless of developmental stage, and regulation of stage-specific events, such as axon elongation, branching, pruning, synapse formation, and synaptic transmission, which is supported by mRNAs whose translation is developmentally regulated. We also found that axonal mRNA translation continues in adulthood, when regulators of neurotransmission and axon survival are locally translated. Bioinformatic analysis of key translational regulators, such as mammalian target of rapamycin complex 1 (mTORC1), fragile X mental retardation protein (FMRP), and adenomatous polyposis coli (APC), reveals that their target mRNAs are translationally co-regulated in a stage-specific manner. In addition, axonally translated mRNAs show extensive isoform diversity, yet only one single isoform is usually translated at any given time and these axonally translated isoforms share common regulatory sequence motifs that promote axonal mRNA translation. Collectively, the results provide direct evidence for the occurrence of developmental stage-specific, compartmentalized mRNA translation in developing and mature CNS axons and provide a deeper understanding of the molecular machinery involved in CNS wiring and maintenance.

## Results

### Retinal RiboTag Labels RGC Axonal Ribosomes In Vivo

**Figure S1:**
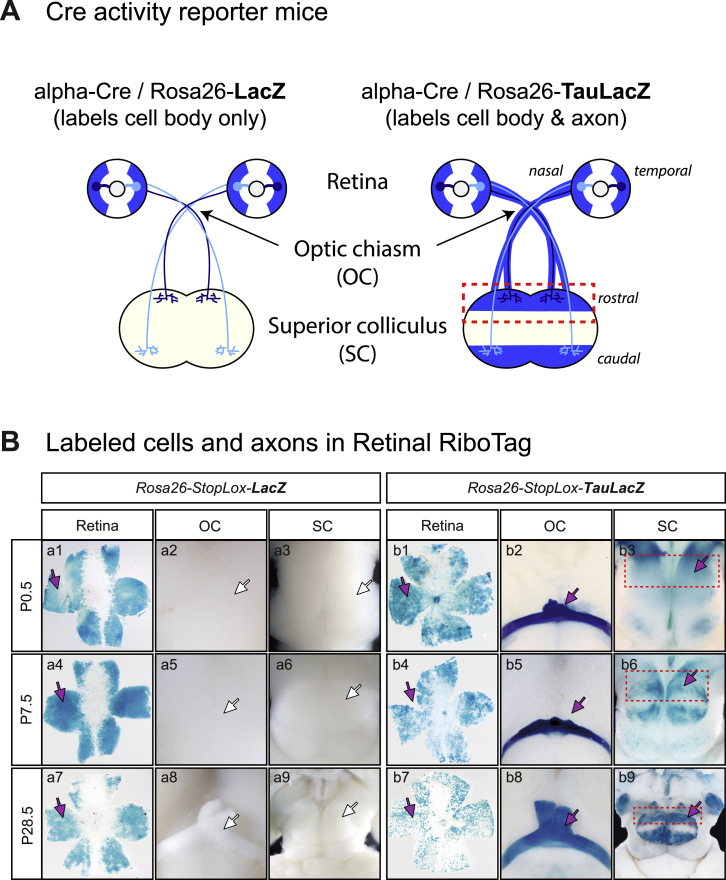
Specific Labeling of Retinal Axons in Retinal RiboTag, Related to Figure 1 (A) Two Cre activity reporter mice were used in this study. The LacZ reporter labels the cell bodies of Cre-positive cells and their progeny, whereas the TauLacZ reporter labels both the cell bodies and axons. (B) X-gal staining of the retina, optic chiasm (OC) and superior colliculus (SC) in alpha-Cre; LacZ reporter gene double positive mice. Cre labels most peripheral neural retinal cells in both mice. No cells in the SC used for TRAP in this study express Cre as evidenced by the lack of X-gal stain in the LacZ reporter SC. Unlike the alpha-Cre; LacZ mice, alpha-Cre; TauLacZ mice show robust staining not only in the cell bodies but also the OC and the SC. The SC, which was used for axon-TRAP, is highly innervated by retinal axons.

In order to isolate mRNAs translated in RGC axon terminals in the SC in vivo, we used the RiboTag knockin mouse line (Sanz et al., 2009), in which Cre-mediated recombination switches the RiboTag allele, which encodes the 60S subunit protein ribosomal protein L22 (RPL22), to the hemagglutinin (HA)-tagged *Rpl22* allele (*Rpl22-HA*). We crossed this mouse with a Pax6-alpha-Cre mouse (Marquardt et al., 2001), which transiently expresses Cre in the neural progenitors in the peripheral retinal primordium, permanently labeling RGCs (Figure 1B; green area in the eye). We confirmed that no resident cells in the SC express Cre by two independent approaches, histological and molecular biological assays (Figures 1C, 1D, and S1; see Supplemental Experimental Procedures). Therefore, the immunopurification of ribosome-mRNA complexes from the dissected SC allows us to profile local translation in axon terminals of RGCs in vivo (axon-TRAP; Figure 1B).

We sought to visualize the labeled ribosomes using an HA antibody. HA immunoreactivity was observed in the distal neural retina (Figures 1E and S1) and the optic nerve head (ONH) (Figure 1E, white box), the soma-free region where RGC axons collect to exit the eye, indicating that the RGC axons do contain HA-tagged ribosomes. To visualize the tagged ribosomes with higher resolution, we employed immuno-electron microscopy (EM). Immuno-gold particles specifically labeled a subpopulation of ultrastructurally identifiable ribosomes (Figure 1F) in the distal neural retina in a Cre-dependent manner (Figure 1G). We successfully detected HA-tagged ribosomes in the axon shaft in the ONH and the optic nerve (ON) (Figures 1H and 1I) and presynaptic terminals in the SC (Figures 1J–1L) at all stages, indicating that HA-labeled endogenous ribosomes are transported to the axon, even in the adult. Together, our histological, molecular biological, and ultrastructural analyses indicate that Retinal RiboTag faithfully labels retinal axonal ribosomes in the SC.

### An Unbiased Identification of the Axonal Translatome

**Figure 2:**
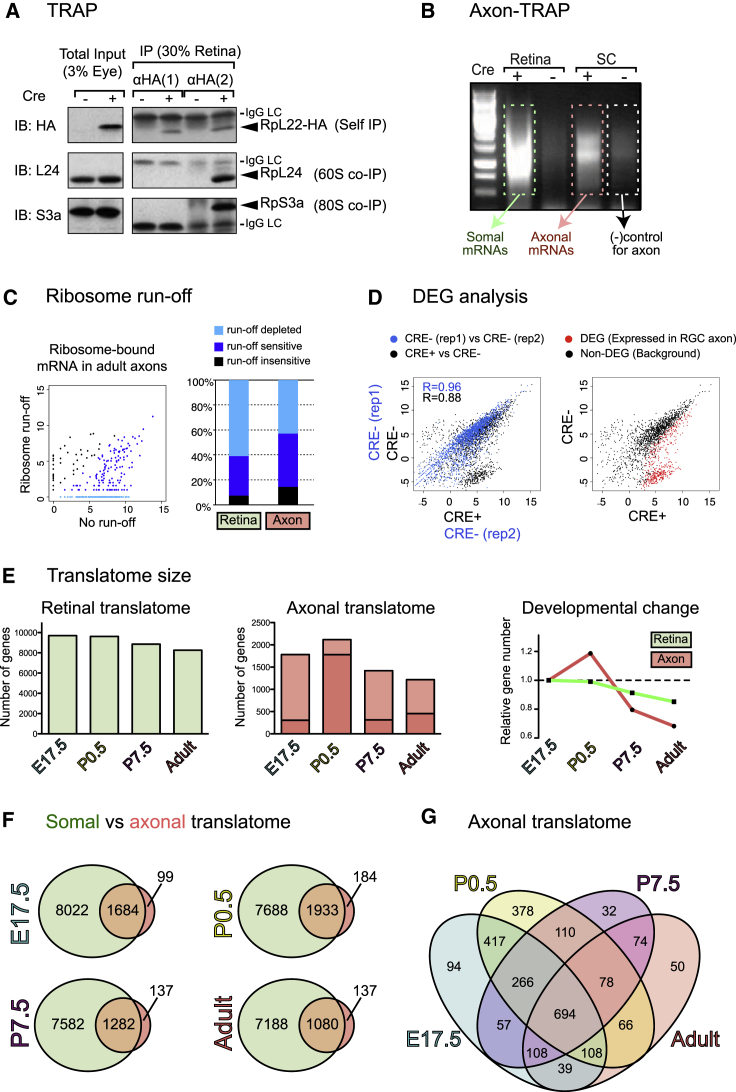
Unbiased Identification of the Axonal Translatome (A) HA-labeled ribosomes were TRAPed by two independent antibodies against HA, and then co-immunoprecipitated ribosomal proteins from 60S (i.e., rpL24) and 40S (i.e., rpS3a) were visualized by western blot. IgG LC, immunoglobulin G light chain. (B) Double-strand cDNAs were made from TRAPed RNAs. (C) Read counts of adult SC samples with or without ribosome run-off. Left panel is a scatter plot of log_2_ (read count+1), and right panel represents the percentage of genes whose read counts were decreased by run-off. (D) A scatterplot of log2 (FPKM) between Cre-positive/-negative (x axis) and Cre-negative axons (y axis) at stage P0.5. (E) Change in numbers of DEGs in the retina and axon. For axon, dark pink indicates DEGs at the corresponding stages. Light pink indicates genes that are DEGs only at that stage. Combined value of orange and peach (union of DEGs) indicates the size of axonal translatome. (F) Somal versus axonal translatomes. (G) Four different axonal translatomes. See also Figure S2.

**Figure S2:**
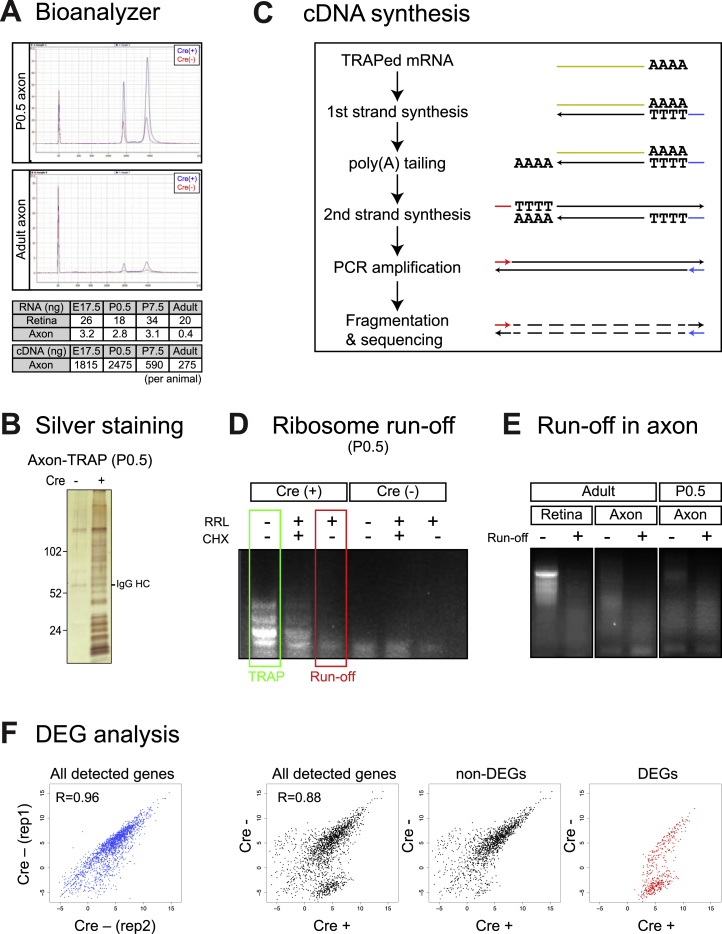
Axon-TRAP, Related to Figure 2 (A) Bioanalyzer analysis of axon-TRAPed mRNA. Lower tables show the amounts of total RNAs and amplified cDNAs for each TRAPed sample. (B) Silver staining of axon-TRAPed protein complexes following SDS-PAGE. (C) Strategy for cDNA synthesis and amplification adapted from the study by Tang et al. (D) Ribosome run-off experiment. The amplified cDNAs from TRAPed mRNAs with or without run-off (P0.5 retina). (E) Retinal and axon-TRAP combined with ribosome run-off. (F) Scatterplots of log_2_ (FPKM) between Cre-positive (x axis) and Cre-negative axons (y axis).

Because the mRNA bound to the labeled axonal ribosomes of RGCs represents only a small fraction of the mRNA in the SC, a major caveat of axon-TRAP is non-specific binding of mRNAs derived from the SC cells to immunoglobulins, Protein G, and magnetic beads. To reduce this background noise, we first optimized the immunopurification protocol before performing axon-TRAP. We estimated that approximately 40% of HA-tagged translating ribosomes could be purified with this optimized protocol (Figures 2A, S2A, and S2B; see Supplemental Experimental Procedures). Successful isolation of axonal ribosomes was confirmed by silver staining (Figure S2B) followed by mass spectrometry (unpublished observation), although RPL22-HA pulled down from the SC was below the level of detection by western blot. To assess the levels of background noise, we compared the levels of cDNAs amplified from TRAPed mRNAs (Figure S2C) between the Cre-positive and -negative littermates. Although axon-TRAP was clearly dependent on Cre and therefore specific, additional amplification led to an increased background (Figure 2B). We took advantage of this background “noise,” reasoning that the Cre-negative samples would control for all the potential causes of false-positive signals that any technical modification could not completely eliminate.

In addition to avoiding any noise in the signal, we also wanted to assure ourselves that the signal came from mRNAs that were actively being translated, because 80S ribosomes can be stalled during translation by translational repressors, such as FMRP (Darnell et al., 2011). In vitro ribosome run-off (see Supplemental Experimental Procedures) decreased the amount of TRAPed mRNAs to the degree that it could not be distinguished from the Cre-negative control (Figure S2D), indicating that the majority of TRAPed mRNA comes from actively translating ribosomes. RNA sequencing analysis showed that we could detect 85% of TRAPed mRNAs isolated from adult axons as being actively translated (Figures 2C and S2E). We use the term “translatome” for ribosome-bound mRNAs in this study, but it should be noted that approximately 15% of these may represent translation-stalled mRNAs.

### Axon-TRAP Identifies Changing Population of Ribosome-Bound mRNAs in Developing and Mature Axons In Vivo

We used axon-TRAP on SCs dissected out at three specific stages during retinotectal development and in the adult: embryonic day 17.5 (E17.5) (elongating); postnatal day 0.5 (P0.5) (branching); P7.5 (pruning); and adult (mature; Figure 1A). To compare the axonal translatome with the somal translatome, we also analyzed the ribosome-bound mRNAs in dissected Cre-positive retina, which contains the cell bodies of RGCs. When we plotted the normalized read count (fragments per kilobase of transcript per million mapped reads [FPKM]) of each mRNA TRAPed from Cre-positive over Cre-negative SC samples, Cre-dependent signals of mRNAs were immediately visible (Figure 2D, left panel, black dots). This was in contrast with the plot with two biological replicates of Cre-negative SC samples, which showed a clear correlation (Figure 2D, left panel, blue dots). To select Cre-dependent mRNAs in an unbiased way, we performed “differential expression analysis” on biological replicates of Cre-positive and -negative samples using NOIseq, which is well suited for quantitative comparisons for independently performed RNA-seq samples (Tarazona et al., 2011; Figures 2D and S2F; see Supplemental Experimental Procedures). We defined these genes as “differentially expressed genes (DEGs)” (Figures 2D and S2F, right panel, red dots; Table S1) and used these for most of the downstream analyses.

**Figure S3:**
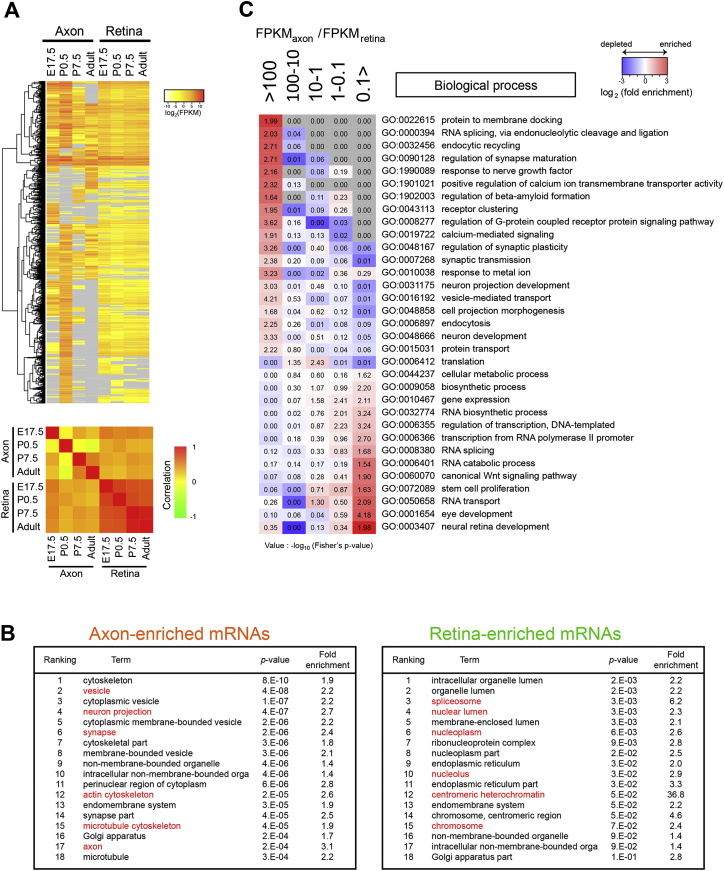
Comparison between the Axonal and the Retinal Translatome, Related to Figure 3 (A) The upper panel shows a heat map of hierarchical clustering on the normalized level of axonal and retinal translation of genes. Each row in the heat map corresponds to a single gene. The color of the heat map represents the log2 (FPKM value) for each gene (gray = not detected). The lower panel shows a heat map of a correlation matrix. (B) Tables showing the ranking of most significantly enriched GO terms in axon-enriched mRNAs and retina-enriched mRNAs. Terms presented in Figure 3B are shown in red. (C) A heat map showing the enrichment of GO terms in the biological process (BP) category. The colors of the heat map represents the log2 value of the fold enrichment for each GO term value (red = enriched, blue = depleted, gray = not detected), and the numbers on the heat map are –log 10 (Fisher’s exact p value) for enrichment.

The total number of axonally translated mRNAs was higher in early stages, peaking at P0.5, and decreased postnatally, whereas mRNAs that are translated within the retinal somas showed little change over the periods examined (Figure 2E), consistent with the amounts of axon-TRAPed cDNAs (Figure S2A). Although previous studies demonstrated that proteins are synthesized in developing axons, it has been controversial whether mature CNS axon terminals also have the ability to synthesize proteins at all, partly because of early studies detecting few or no ribosomes in mature axons (references in Piper and Holt, 2004). However, the presence of DEGs, approximately 85% of which were confirmed as being translated (Figures 2C and S2E) and ribosomes (Figures 1I and 1L) in adult axons indicates that axonal mRNA translation persists in adult CNS axons. The axonal translatome of RGCs is largely an evolving subset of the significantly larger somal translatome (Figure 2F), confirming that axon-TRAPed mRNAs originate from RGC neurons. Unlike the somal translatome (Figure S3A), however, the axonal translatome showed extensive developmental regulation (see detailed analysis below) with only 694 out of 2,576 (27%) mRNAs translated at all stages (Figures 2G and S3A), indicating that the axonal translatome is not due to the simple passive diffusion of translated mRNAs from the soma.

### Axon-TRAPed mRNAs Encode Axon-Specific Proteome

**Figure 3:**
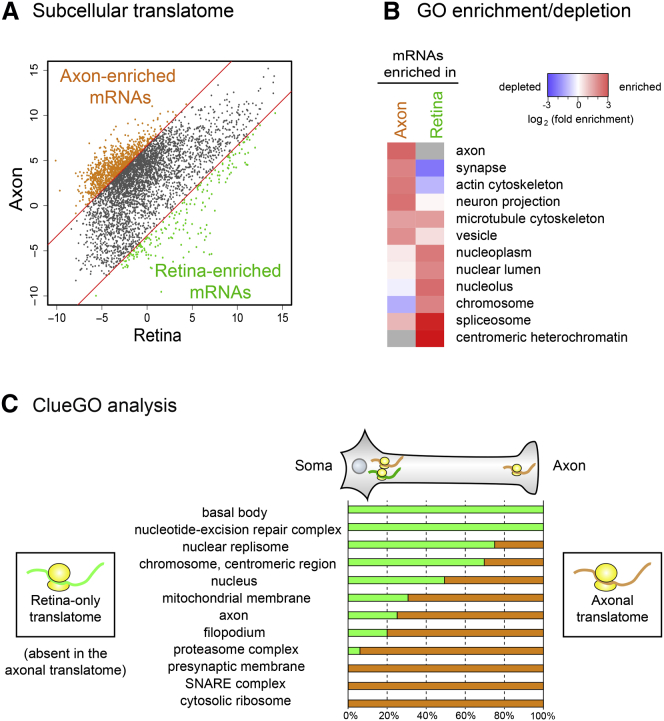
Comparison between the Axonal and Retinal Translatomes (A) Normalized mRNA levels (log_2_(FPKM)) between the axonal (y axis) and retinal (x axis) translatome at stage P0.5. Axon- and retina-enriched population were defined when FPKM_axon_/FPKM_retina_ > 100 and <0.1, respectively. (B) GO terms in the cellular component category. More detailed lists are in Figure S3B (gray, not detected). (C) ClueGO analysis. The left axis indicates the parental GO terms. The percentage of daughter GO terms associated with somal and axonal translatome is presented. See also Figure S3.

To discover which classes of mRNAs are preferentially translated in the axon, we performed a gene ontology (GO) enrichment/depletion analysis for genes whose translation level is significantly higher (>100-fold difference) in the axon than in the retina (Figure 3A; “axon-enriched mRNAs”). Reassuringly, analysis with the cellular component category showed that axon-enriched mRNAs generally encode proteins that are already known to function in axons, growth cones, and synapses (Figures 3B and S3B). In contrast, mRNAs encoding nuclear proteins (e.g., modifier of chromatin structures) are depleted from axonal translatome. GO terms selectively enriched in the axonal translatome included those involved in vesicle-mediated transport and calcium-mediated signaling (Figure S3C), suggesting that these processes, which play key roles in the distal axon, may be regulated by local mRNA translation.

To explicitly compare axonal and somal translatomes, we used ClueGO software, which reports how many genes in each cluster are assigned with specific GO terms. We compared 2,576 axonally translated mRNAs (“axonal translatome”) with the same number of mRNAs that are most abundant in the somal translatome but absent in the axonal translatome (“retina-only” translatome). We found that synapse- and axon-related GO terms were generally associated with the axonal translatome, whereas the retina-only translatome was enriched with basal body and nuclear GO terms (Figure 3C). These results indicate the presence of mechanisms for selecting specific mRNAs for axonal translation.

### Axonal Translatome Changes from Axon Elongation to Neurotransmission during Development

**Figure 4:**
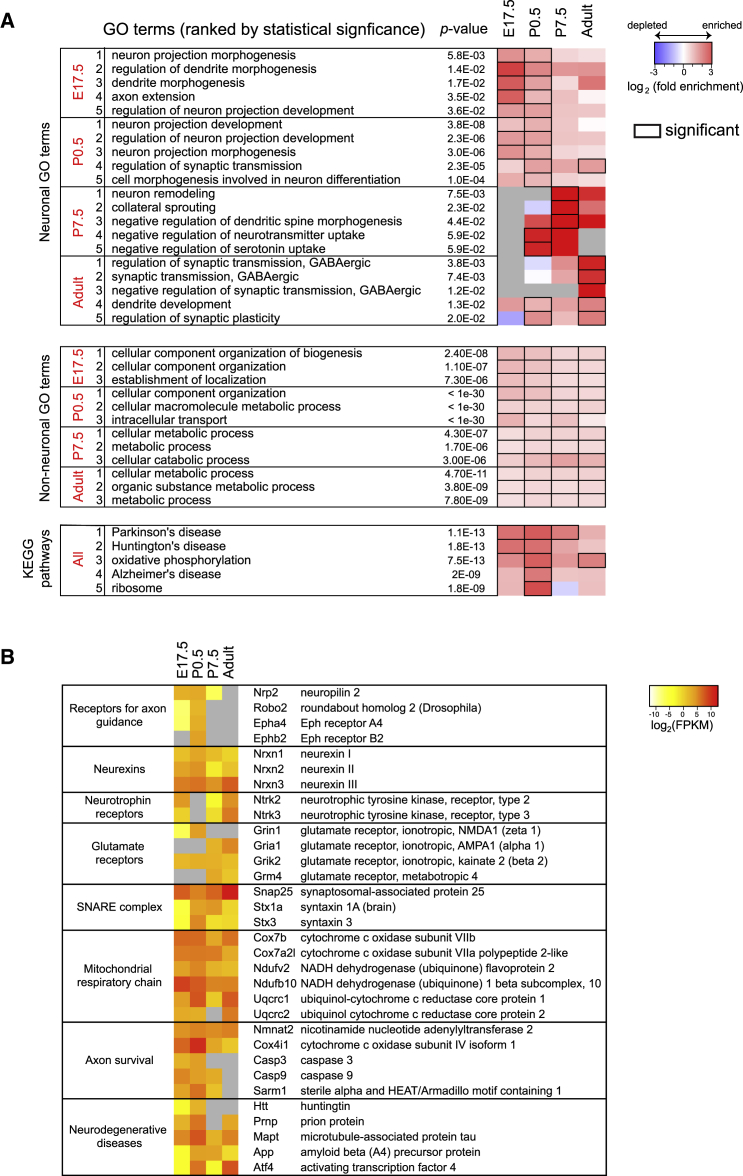
Developmental Changes of Translated Genes in RGC Axons (A) Enriched GO (biological process) terms and Kyoto Encyclopedia of Genes and Genomes (KEGG) pathways for axonally translated genes (grey, not detected), sorted by significance for each stage (Fisher’s exact test). The enrichment was analyzed by topGO (Table S3). Statistically significant cells are marked by black squares. (B) Normalized levels of axonal translation for selected genes (gray, not detected). See also Figure S4.

**Figure S4:**
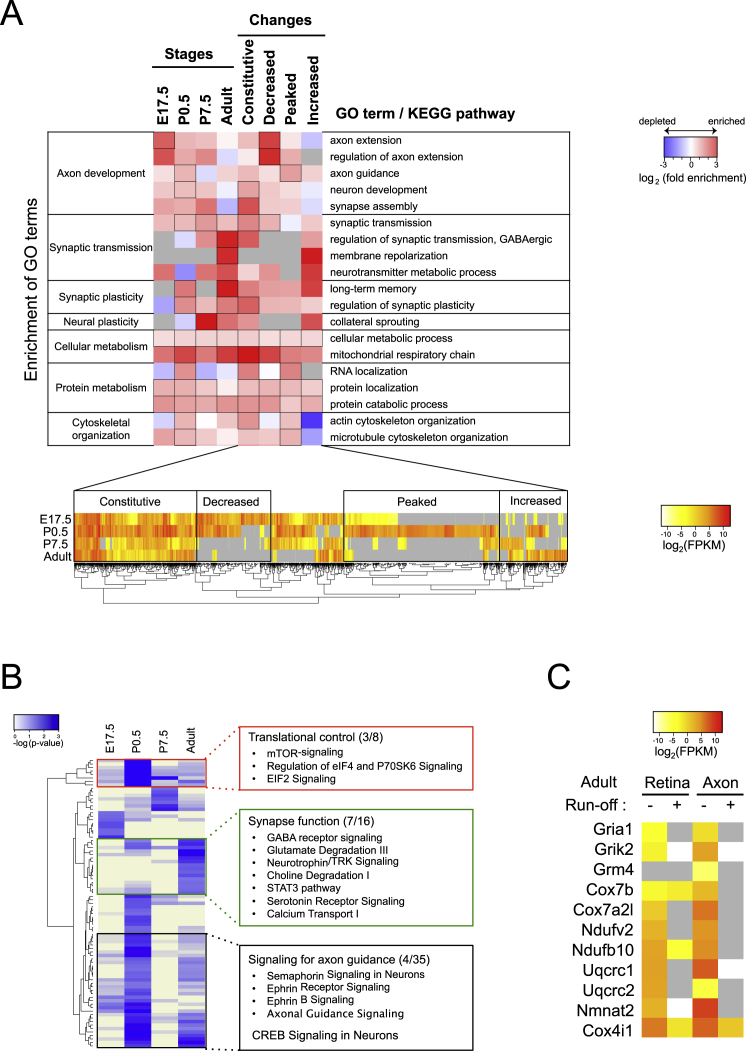
Gene Set Enrichment Analysis Describing the Developmental Changes of Translated Genes in RGC Axons, Related to Figure 4 (A) The upper heat map displays the enrichment of GO terms or KEGG pathways for axonally translated genes. Each row in the heat map corresponds to a single GO term. Genes are clustered either by stage-specific expression (“stages”) or hierarchical clustering (lower heat map) according to their developmental changes (“changes”). (B) Ingenuity pathway analysis (IPA) to identify canonical pathways associated with the axonal translatome. Each row represents a single pathway (blue, enriched). The right panel shows lists of pathways extracted from each cluster. (C) A heatmap showing the log_2_ (read count) for adult samples with and without ribosome run-off. Each row in the heat map corresponds to a single gene.

To correlate the local translation with the stage-specific events in axon development, we performed a GO-based analysis for genes that are translated in axons at each developmental stage using 455 neuron-related GO terms (Table S2). The translatome in younger axons (E17.5 and P0.5) was highly enriched with axon-development-related GO terms, including “neuron projection morphogenesis,” whereas that of older axons (adult) was enriched with synaptic-transmission-related GO terms, such as “synaptic transmission” (Figures 4A and S4A; Table S3). The ingenuity pathway analysis (IPA) for canonical signaling pathways also suggests that synapse function was most highly regulated in adult axons (Figure S4B). This result suggests that axonal mRNA translation continues in the mature CNS of mammals in vivo and may regulate presynaptic function. We found that a number of genes, which are robustly translated in adult axons, encode glutamate receptors and neurotrophin receptors (Figures 4B and S4C), some of which are known to regulate synaptic transmission in the presynaptic compartment (Pinheiro and Mulle, 2008). Furthermore, key components of the trans-SNARE complex, which mediates neurotransmitter exocytosis, are highly translated in mature axons (Figure 4B), suggesting that their local translation plays a role in supporting the core machinery of neurotransmission in presynapses.

Intriguingly, translation of receptors for axon guidance molecules peaks around birth (P0.5) and falls off thereafter (Figures 4B and S4A). Because this is when interstitial branches arise from axon shafts in a topographically biased manner to connect with targets (Figure 1A), stage-specific synthesis of these receptors in the RGC axon may help to fine-tune topographically biased branching. We also noted that the GO terms “neuron remodeling” and “collateral sprouting” were among most enriched in the pruning stage (P7.5). Genes that function in synapse assembly, which include neurexins and presynaptic cell adhesion molecules, were translated in all axons (Figures 4B and S4A).

### The Axonal Translatome Changes from Degenerative to Survival Modes at the End of Development

Axon survival is regulated through at least two pathways: by maintaining axonal protein/energy homeostasis and by inhibiting a destruction program mediated by SARM1. *Sarm1*, which initiates a soma-independent axon destruction program by counteracting *Nmnat* function (Gerdts et al., 2015), is highly translated in developing, but not in adult, axons (Figures 4B and S4C). The same pattern of local translation was observed for caspases, whose local action mediates axon dynamics and developmentally controlled branch destruction (Campbell and Holt, 2003, Campbell and Okamoto, 2013, Simon et al., 2012). These results suggest that developing (arborizing) axons synthesize the components of axon degeneration pathways, perhaps in highly restricted subcellular compartments within the axon, for the selective withdrawal of branches, whereas adult axons shut them off to maintain mature neural connections for long periods of time.

GO terms related to mitochondrial and homeostatic functions, such as “cellular metabolism” and “mitochondrial respiratory chain,” were enriched at all stages, supporting the previous finding that axonal mRNA translation supports mitochondrial function and is required for axon survival (Figures 4A and S4A; Cosker et al., 2016, Hillefors et al., 2007, Yoon et al., 2012). The survival of a neuron whose axon reaches its correct target is regulated by retrograde transmission of a survival signal from the axon terminal, which turns on a transcriptional program for cell survival (Riccio et al., 1997). Previous studies showed that axonal synthesis of transcription factors, such as neurotrophin-induced synthesis of CREB (Cox et al., 2008) and SMAD1/5/8 (Ji and Jaffrey, 2012) and axon-injury-induced synthesis STAT3 (Ben-Yaakov et al., 2012), regulates cell survival during development and in adulthood. Indeed, our IPA analysis revealed that components of these nuclear signaling pathways, including CREB and STAT3 signaling, are enriched in adult axons (Figure S4B). Therefore, our results suggest that local translation promotes survival of mature axons both by supporting mitochondrial function and actively generating survival signals.

Pathological axon degeneration in neurodegenerative diseases has been associated with impaired axonal translation (references in Jung et al., 2012). A KEGG pathway enrichment analysis showed a significant over-representation of genes linked to neurodegenerative diseases, such as Alzheimer’s, Parkinson’s, and Huntington’s diseases (Figure 4A). In particular, we detected robust axonal translation of huntingtin (*Htt*), Prion protein (*Prnp*), microtubule-associated tau (*Mapt*), and amyloid beta precursor protein (*App*), whose aggregates are strongly associated with neurodegenerative disorders (Figure 4B), suggesting a possible connection between axonal translation and neurodegeneration involving protein aggregation. Intriguingly, activating transcription factor 4 (Atf4), whose excessive axonal translation spreads Alzheimer’s disease pathology across the brain (Baleriola et al., 2014), is also axonally translated at all stages tested. These results support the idea that dysregulated axonal translation may be an underlying cause of neurodegenerative diseases (Jung et al., 2012).

### Targets of mTORC1, FMRP, and APC Show Translational Co-regulation in a Stage-Specific Manner

**Figure 5:**
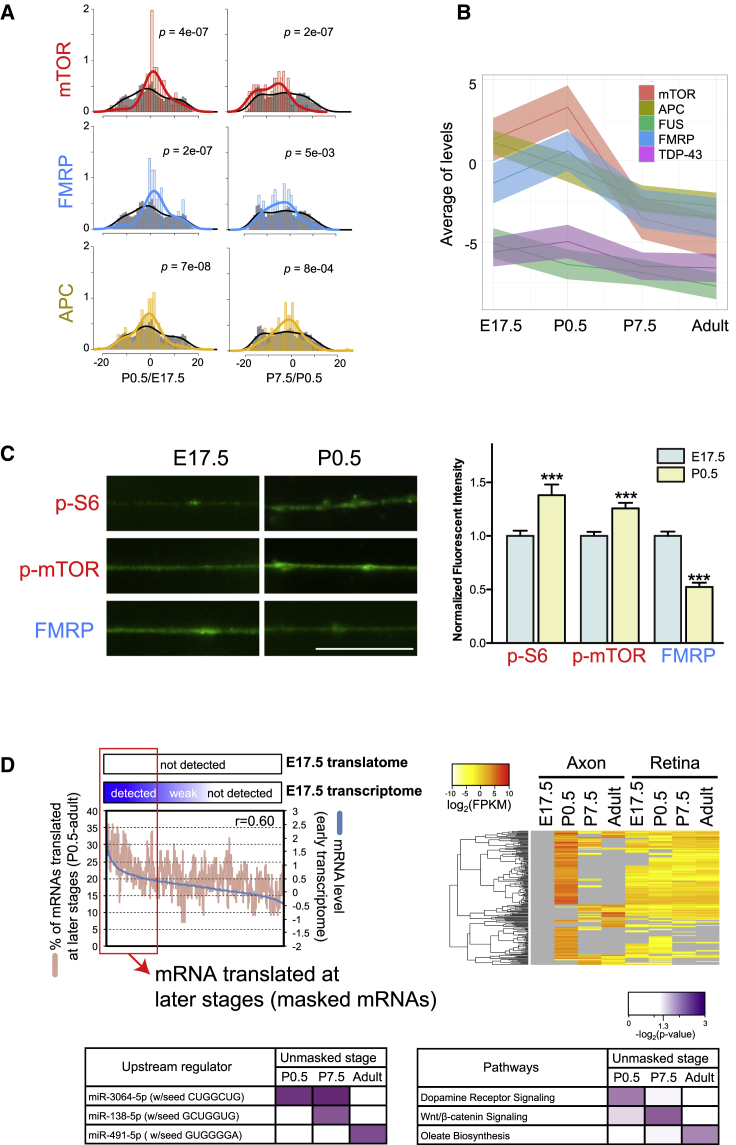
*Trans*-Acting Elements that Regulate the Axonal Translatome (A) Density plots of the change in FPKM values of axonal translatomes during two consecutive developmental stages (log2(stage A [FPKM]/stage B [FPKM]); gray, distribution of all genes; colors, distribution of target genes) with p values (Kolmogorov-Smirnov test). (B) Average log2 (FPKM) values of target genes (mean ± 95% confidence interval). (C) Representative immunofluorescence images (left) and their quantification (right; mean ± SEM). ^∗∗∗^p < 0.001, Mann-Whitney test. The scale bar represents 10 μm. (D) Relationship between transcript abundance of genes not detected in E17.5 axonal translatome (read count = 0) and probability of their translation at later stages (upper left: blue line, mRNA level in transcriptome; red line, moving averages of percentage of genes detected at any of three later stages over a window size of 100 genes; r, Pearson correlation coefficient). The upper right and lower heatmaps show mRNA abundance in the translatome and enriched regulators/pathways, respectively. See also Figure S5.

**Figure S5:**
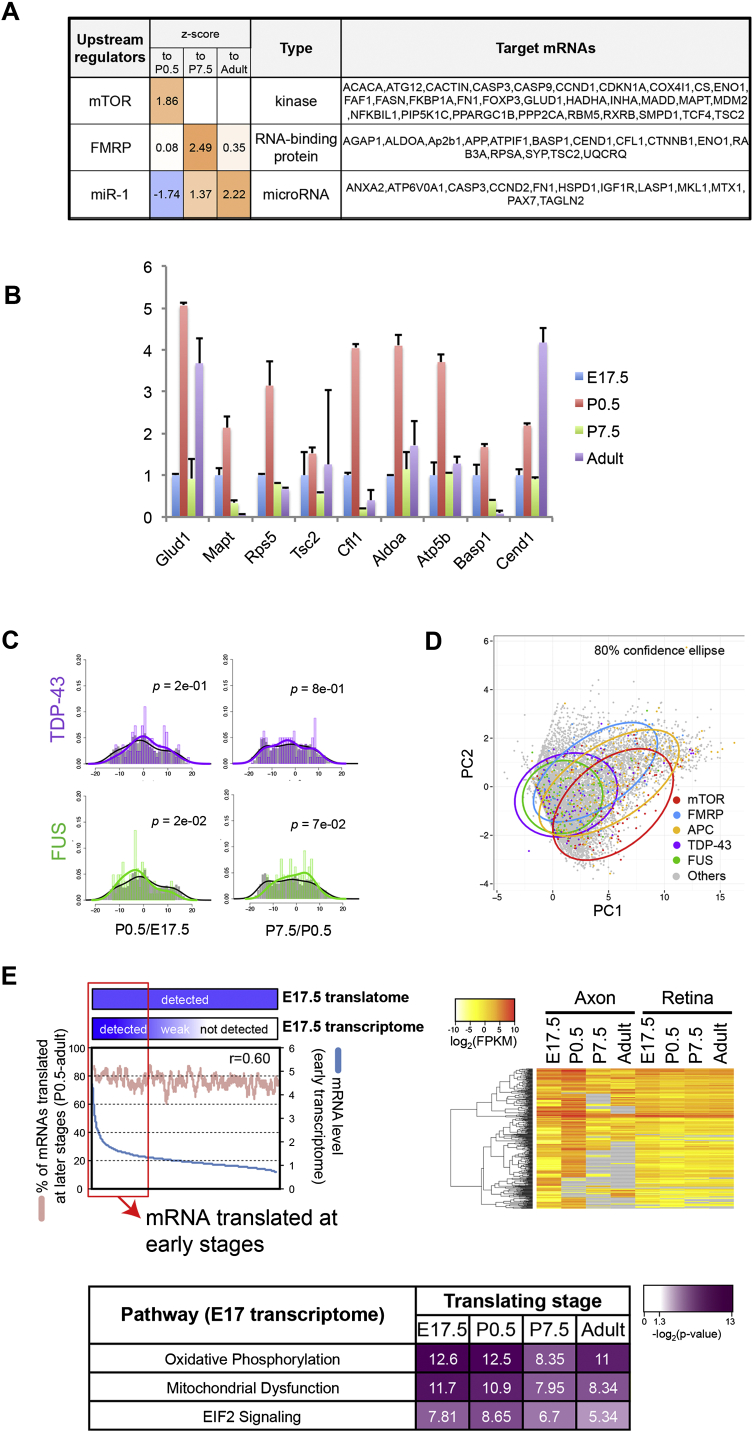
Analysis of *trans*-Acting Elements that Regulate the Axonal Translatome, Related to Figure 5 (A) Ingenuity pathway analysis (IPA) to identify upstream regulators associated with the axonal translatome. The abundance of each mRNA between two consecutive stages was represented as the ratio (ratio > 1 indicates increase in translation). The coordinate change in the translation ratios was calculated as the activation z-score. A positive z-score indicates that the translational regulator is expected to be activated. (B) Bar graph representing the fold change in levels of axonally translated genes. The mRNA levels in the axonal translatome were quantified by qRT-PCR (normalized by TRAPed cDNA for each stage). (C) Density plots showing the distribution of changes in FPKM values for the axonal translatome during two consecutive developmental stages with p values (Komogorov-Smirnov test). The values are calculated as follows: log2 (stage A(FPKM)/stage B (FPKM)). The distributions of target genes in pathways, which are indicated by colored lines, are overlapped with non-target genes represented by gray lines. (D) A scatter plot of the Principal Component Analysis (PCA) based on normalized read counts in the axonal and retinal translatome from four different stages. Data were plotted using the first two Principal Components (PCs), which explained up to 73.2% of the total variance. (E) Relationship between transcript abundance of the genes in E17.5 transcriptome, which were detected in E17.5 axonal translatome, and probability of their translation at later stages (blue line, mRNA level in transcriptome; red line, moving averages of percentage of genes detected at any of three later stages over a window size of 100 genes; r, Pearson correlation coefficient). The right heatmap shows mRNA abundance in the translatome (left) and enriched pathways (right).

We have shown that the axonal translatome is dynamically regulated during development, and this raises the important question of how axonal translation is controlled by upstream signaling pathways. To investigate this, we performed IPA upstream regulator analysis, which is based on published data of gene knockdown or knockout studies where protein products were measured when translational regulator function was impaired. mTORC1 activity was predicted to peak in actively wiring axons, as its target mRNAs showed a steep increase at P0.5 (Figure S5A), consistent with previous studies demonstrating that axonal mRNA translation is regulated by mTORC1 (Campbell and Holt, 2001) and required for axon branching (Spillane et al., 2013). In contrast, the activity of FMRP was predicted to peak later at P7.5, because its target mRNAs (whose translation is repressed) showed a coordinate decrease in translation in mature axons (Figures 5A, 5B, S5A, and S5B). This result suggests that the translational brake mediated by FMRP is utilized in maturing CNS axons as in dendrites (Bagni and Greenough, 2005, Darnell and Klann, 2013). Consistent with this result, known targets of FMRP and mTORC1 in the axonal translatome showed clearly different translational patterns from the non-target mRNAs: their translation increased at P0.5 (Figure 5A, left panel, red and blue; median shifts right) and decreased at P7.5 (right panel). Another intriguing translational regulator was APC, which was recently shown to regulate microtubule assembly and axonal growth by local translation (Preitner et al., 2014). Our analysis indicates that the translation of APC target mRNAs is highest in the youngest axons (E17.5) and steadily decreases thereafter (Figures 5A and 5B), consistent with the primary role of microtubule assembly in axon growth. In contrast, the targets of TDP-43 and FUS, well-known neuronal RNA-binding proteins (RBPs), showed a distribution not significantly different from the total axonal translatome (Figures 5B and S5C), although the possibility remains that TDP-43 and FUS regulate stage-independent axonal mRNA translation. A principal-component analysis (PCA) also showed clear separation of the mTORC1, FMRP, and APC targets from the rest of the axonal translatome (Figure S5D). MicroRNAs (miRNAs) make up another class of translational regulators that function in the axon (Sasaki et al., 2014). We found that the translation of miR-1 target mRNAs decreases as the axon matures, suggesting that miR-1 abundance and/or activity increases during RGC axonal development (Figure S5A).

We took an independent approach to investigate the possibility of developmental-stage-dependent regulation of mTOR and FMRP signaling in RGC axons. We measured the abundance of phosphorylated mTOR (p-mTOR) and S6 (p-S6) in cultured primary mouse RGC axons by quantitative immunofluorescence (QIF), which positively correlate with mTORC1 activity (Copp et al., 2009, Laplante and Sabatini, 2012; Figure 5C). We found that they increased between E17.5 and P0.5, supporting our hypothesis that mTORC1 activity rises in RGC axons during this period. In contrast, the level of FMRP decreased in the same period, in accordance with our model that the translational brake is weakened in P0.5 axons (Figure 5C).

To gain more insight into mRNA-specific translation in the axon, we compared the RGC axon transcriptome of E17.5 (Zivraj et al., 2010) to the axon translatome at the same stage. We analyzed the genes that are detected in the transcriptome, but not in the translatome, because this group may contain candidates for translationally repressed (“masked”) mRNAs. We found that a significant portion of these candidates was translated at the three later stages tested (P0.5, P7.5, or adult) because their levels in the transcriptome correlated with the probability for translation at later stages (Figure 5D). This suggests the possibility that the mRNAs, which are present in high abundance, but not translated, are being stored for translation in later stages. In contrast, the genes that are present both in the young transcriptome and translatome did not show this trend (Figure S5E).

Strikingly, mRNAs that are “unmasked” at the same stage encode various components of specific signaling pathways (Figure 5D). For example, components of dopamine receptor signaling, WNT/β-catenin signaling, and the oleic acid biosynthesis pathway were specifically unmasked in P0.5, P7.5, and adult axons, respectively. Additionally, as noted above, mRNAs that are unmasked at the same stage share common translational regulators (Figure 5D). Together, these results show that functionally coherent sets of mRNAs are coordinately translated in the axon by shared upstream regulators.

### Alternative Splicing Generates mRNA Isoform Diversity in the Axon

**Figure 6:**
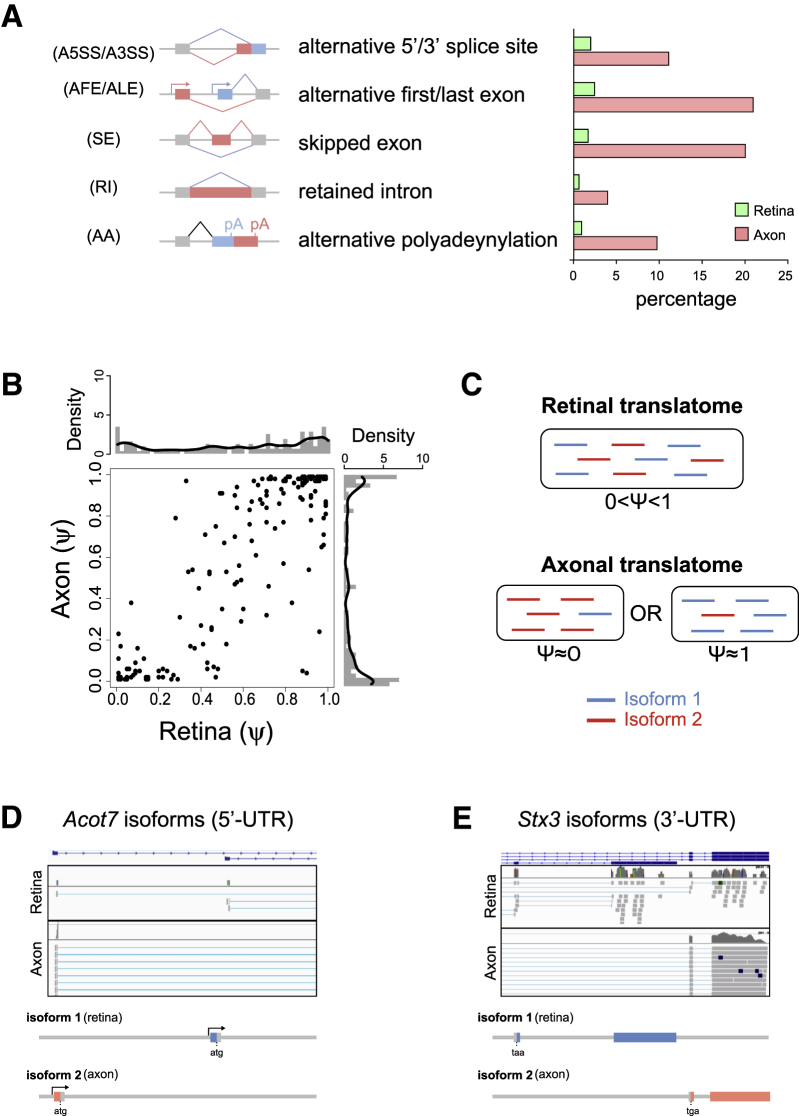
Alternative Splicing Generates High Isoform Diversity in Axons (A) Percentage of genes with alternative events from all axonally translated genes. Alternative events are classified into five different classes depicted in the left panel. (B) Scatter and density plots for the distribution of percentage spliced in (Ψ) values between the retina (x axis) and the axon (y axis). (C) Model for biased distribution of Ψ values in the axon. The comparison of two isoforms suggests that one of two isoforms is predominant in the axon. (D and E) The sequence reads on *Acot7* and *Stx3* loci visualized with integrative genomics viewer (IGV). The histograms show the depth of the reads displayed at each locus. The retinal isoforms are detected only in the retinal translatome, whereas the axonal isoforms are detected both in the axonal and retinal translatomes. See also Figure S6.

**Figure S6:**
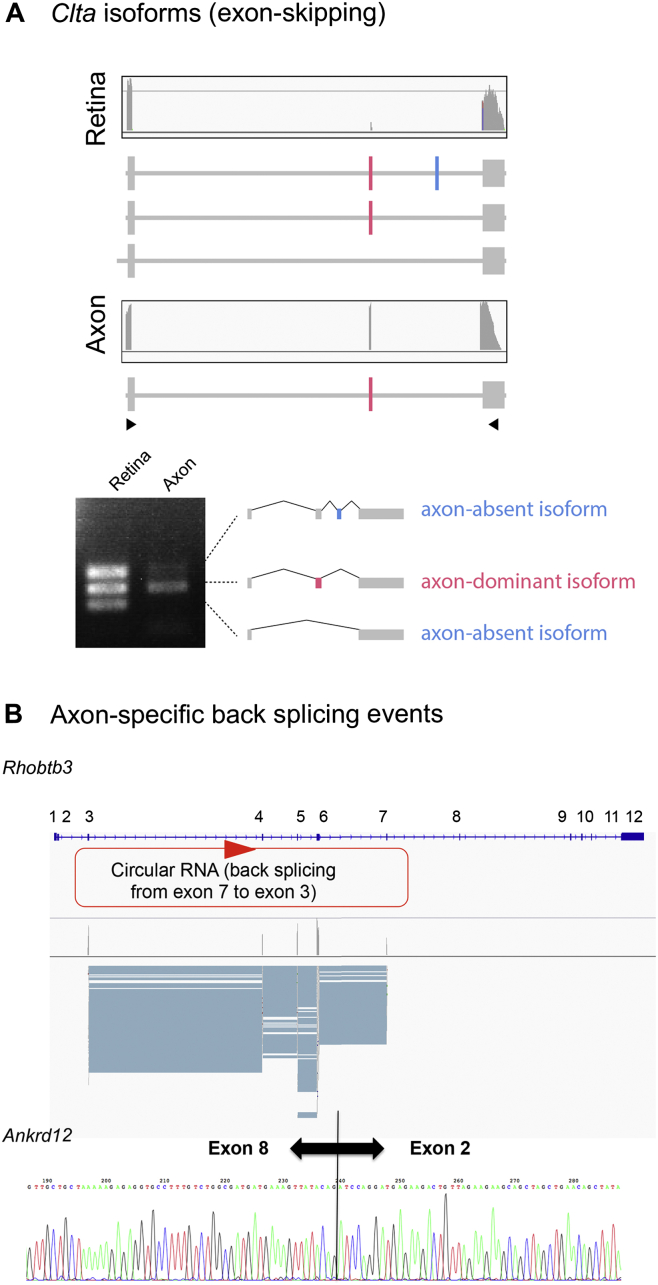
Analysis for Alternative Isoforms and *cis*-Acting Elements, Related to Figure 6 (A) Sequence reads (gray bars) mapped on the *Clta* gene. The mapped reads are visualized with Integrative Genomics Viewer (IGV). (B) Sequence reads (gray bars) mapped on the *Rhobtb3* (upper panel). Sanger sequencing of RT-PCR fragment of the *Ankrd12* mRNA (lower panel).

Post-transcriptional RNA processing events, including alternative splicing, are widely used to control gene expression in neurons. To assess whether these regulate local mRNA translation, we analyzed the mRNA isoforms on mapped sequence reads using MISO software (Katz et al., 2010). Intriguingly, the axonal translatome showed more-extensive diversity of mRNA isoforms than the somal translatome (Figure 6A). To address the possibility of isoform-specific axonal translation, we selected 164 alternative events that produce two isoforms both in the axonal and retinal translatomes. Then, we calculated the “percentage spliced in” (PSI or Ψ) values, which represent the fraction of the longer isoform (Katz et al., 2010). Ψ _retina_ was uniformly distributed (0 < Ψ < 1), indicating that there is no clear bias in translational efficacy (Figures 6B and 6C). However, Ψ _axon_ was biased to the two extremes (i.e., Ψ = 0 or Ψ = 1), indicating that only one of the two isoforms is selectively translated in the axon (Figures 6B and 6C). Notable examples are *Acot7*, an acyl-coenzyme A (CoA) thioesterase gene required for lipid biosynthesis and neuron survival (Ellis et al., 2013); *Syntaxin 3* (*Stx3*), a SNARE component gene; and *Clta*, a clathrin light chain A gene, which show clear axon-specific usage of first, last, and internal exons, respectively (Figures 6D, 6E, and S6A). Intriguingly, axon-specific isoforms of *Acot7* and *Stx3* encode proteins with slightly different amino acids at the N and C termini, respectively (see gene models in Figures 6D and 6E), suggesting that alternative splicing may couple axon-specific protein isoforms with a unique sequence tag in the UTR.

Unexpectedly, we detected a number of back splicing events for three genes (*Rhobtb3*, *Ubn2*, and *Ankrd12*), which indicate the potential presence of circRNAs in the axonal, but not in the retinal, translatome, and we could detect these mRNAs by RT-PCR of unamplified axonal translatome (Figure S6B). Although previous studies suggested that the circRNAs are not translated (Guo et al., 2014), our result raises the possibility that the ribosomes can associate with circRNAs in axons. However, further studies are needed to address whether proteins are actually synthesized from these circRNAs.

### *Cis*-Regulatory Elements Couple Alternative Splicing with Axonal Translation

**Figure 7:**
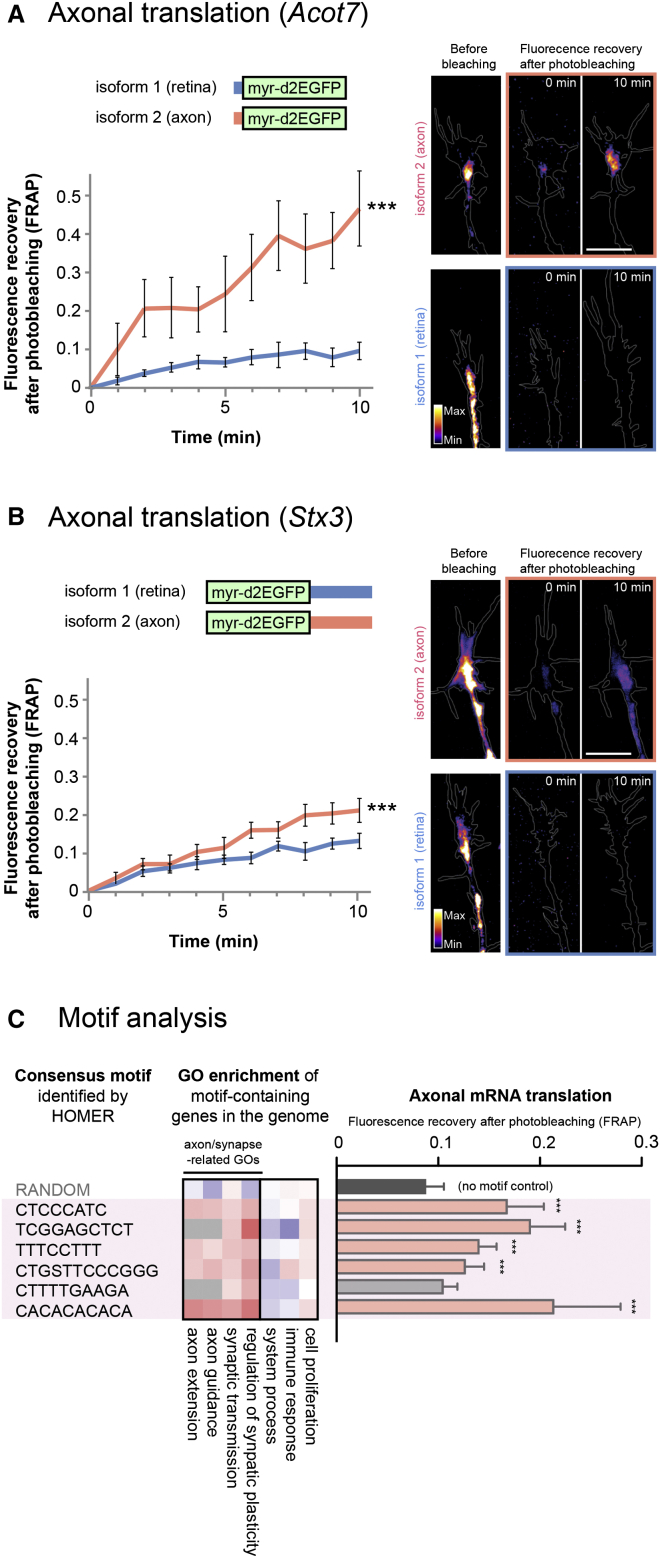
*Cis*-Regulatory Elements Link Alternative Splicing to Axonal Translation (A and B) Axon- and retina-specific *Acot7* and *Stx3* UTR isoforms fused with myr-d2EGFP were expressed in cultured RGCs (*Xenopus*). Quantification of fluorescence intensity after photobleaching (FRAP) revealed axon-specific isoforms of *Acot7* (A) and *Stx3* (B) markedly increase axonal translation of the myr-d2EGFP reporter construct compared to retina-specific UTR counterparts. Data at each 1 min time point represent the mean fraction of recovery relative to pre- and post-bleach levels ± SEM (n = 9 and 10 for axon and eye-specific 5′ UTR of *Acot7*, respectively; n = 14 and 14 for axon and eye-specific 3′ UTR of *Stx3*, respectively). ^∗∗∗^p < 0.0001; two-way ANOVA. FRAP signal recovery was abolished by 40 μM anisomycin (10 min post-photobleach: Acot7 axon-isoform + anisomycin 0.064 ± 0.028; *Stx3* axon-isoform + anisomycin 0.085 ± 0.026). Representative images of RGC axonal growth cones showing fluorescent recovery after photobleaching for each reporter construct are shown (right). The scale bars represent 10 μm. (C) GO enrichment analysis for entire genome containing axon-specific sequence motifs associated with alternative exons (S: G or C) and their relative efficiency in axonal mRNA translation using myr-d2EGFP reporter constructs. Significance of FRAP recovery curves were compared to no UTR control across 10 min (n ≥ 10 for each construct). Statistical significance of FRAP compared to the no-UTR control was tested across all time points (1–10 min) using a two-way ANOVA (^∗∗∗^p < 0.0001 compared to no-UTR control). For representative purposes, the mean fluorescence recovery at 10 min post-photobleaching is shown. Error bars represent SEM. See also Figure S7.

The dominance of a single alternative exon in axons suggests that axonal mRNA translation might be mechanistically linked to alternative splicing. We focused on the axonally enriched mRNAs with an alternative first or last exon because 5′ and 3′ UTRs generally contain localization signals (references in Jung et al., 2012; Figures 6D and 6E). In order to investigate whether the axon-specific exons are sufficient to promote axonal mRNA transport and translation, we used a diffusion-limited, membrane-targeted EGFP (myr-d2EGFP), which is a faithful reporter of local protein synthesis in dendrites (Aakalu et al., 2001) and in axons (Andreassi et al., 2010, Cox et al., 2008). We fused the axon-specific or axon-absent (retina-restricted) alternative exon of each gene to myr-d2EGFP so that a reporter mRNA containing each motif would be generated in cells (Figures 7A and 7B). To test these reporters in RGCs, the same cell type from which they were identified, we used *Xenopus* primary retinal cultures (Campbell and Holt, 2001), which are amenable to screening multiple motifs. We confirmed that alternative usages of the 5′ and 3′ UTRs of *Acot7* and *Stx3*, respectively, are conserved between mouse and *Xenopus* (Xenbase and UCSC genome browser). Fluorescence recovery after photobleaching (FRAP) was monitored in the growth cones of cultured RGCs at 1-min intervals for 10 min. Remarkably, the axon-specific isoforms showed rapid and robust FRAP signals, whereas the retina-specific isoforms did not (Figures 7A and 7B). These results indicate that axon-specific exons of *Acot7* and *Stx3* are sufficient to promote axonal mRNA translation (Figures 7A and 7B).

**Figure S7:**
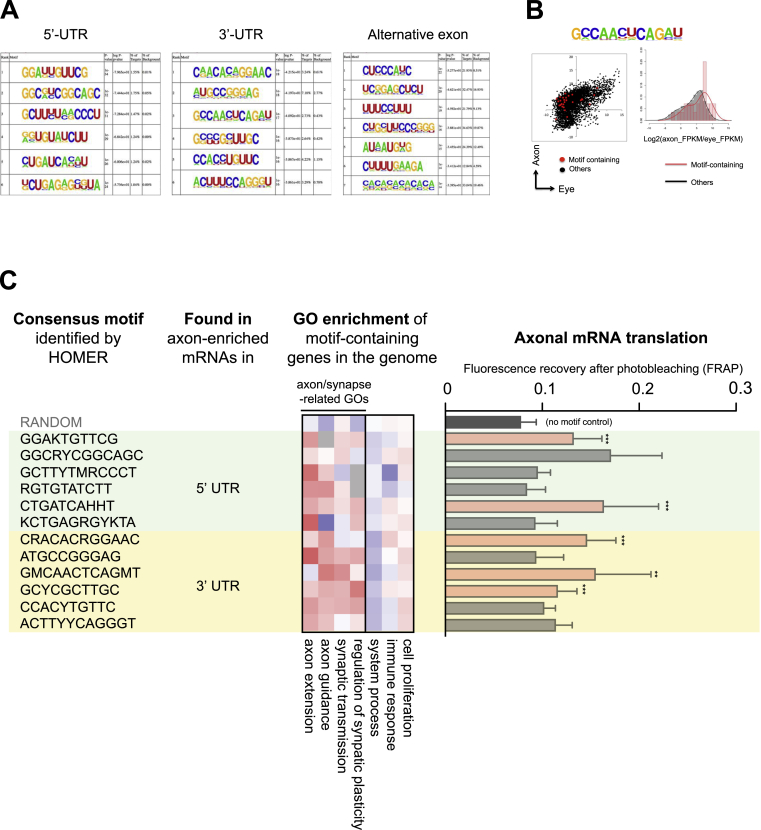
*Cis*-Regulatory Elements for Axonal Translation, Related to Figure 7 (A) Lists of sequence motifs enriched in 5′UTRs, 3′UTRs and alternative exons of axon-enriched mRNAs / exons. (B) An example of axon-enriched motifs. The scatterplot compares the normalized mRNA levels (log2(FPKM)) between the axonal (y axis) and the retinal (x axis) translatome at stage P0.5 for genes with (red dots) and without (black dots) the motif. The density plot shows the distribution of log2 (axon (FPKM) / retina (FPKM)). (C) GO enrichment analysis for entire genome containing axon-specific sequence motifs (K: G or T; R: A or G; Y: C or T; M: A or C; R: A or G; and H A or C or T) and their relative efficiency in axonal mRNA translation measured by fluorescence recovery after photobleaching (FRAP) of motif-containing reporter constructs (myr-d2EGFP). Several axon-specific motifs were able to promote mRNA translation in the growth cone relative to a control myr-d2EGFP construct without a UTR. Statistical significance of FRAP compared to the no-UTR control was tested across all time-points (0-10mins) using a two-way ANOVA (from the top bar, n = 16, 5, 5, 7, 8, 7, 3, 8, 8, 8, 5, 8, and 6, respectively). For representative purposes, the mean fluorescence recovery at 10 min post-photobleaching is shown. Error bars represent SEM. ^∗∗^p < 0.01, and ^∗∗∗^p < 0.001 compared to no-UTR control.

We next investigated whether axon-specific exons might contain “generalizable” motifs responsible for axonal mRNA translation. We searched for common sequence elements that are enriched in axon-specific alternative exons (Figure 7C) and in the 5′ and 3′ UTRs in constitutive exons (Figures S7A and S7B) of axon-enriched mRNAs (Figure 3A). To understand the potential function of identified sequence elements, we searched for genes that contain these elements in the entire mouse genome. Remarkably, the element-containing genes generally encode regulators of axon and synapse function (Figures 7C and S7C). Strikingly, five of six motifs identified from alternative exons and five of twelve motifs in constitutive exons of axon-enriched mRNAs showed a significant FRAP signal at 10 min, indicative of increased axonal mRNA translation of a reporter mRNA when incorporated in the 5′ or 3′ UTR as in Figure 7B (Figures 7C and S7C). These results suggest the potential links between the sequence elements and axonal mRNA translation and thus provide further insight into the mechanisms underlying the selective and dynamic nature of the axonal mRNA translation.

## Discussion

Here, we developed a mouse model of axon-TRAP to isolate mRNAs translated in the distal axon of RGCs in vivo and performed a genome-wide survey of the axonal translatome at critical time points during the assembly of visual circuitry and in adulthood. The axonal translatome is generally a subpopulation of its somal counterpart but is enriched in genes with axon-specific roles. We found that broadly two classes of local translatomes exist in the distal axon: one being constitutively translated and the other being developmentally regulated. The former generally encodes the regulators of protein and energy homeostasis, and the latter encodes proteins required for stage-specific events, such as axon elongation, axon branching, synapse formation, and synaptic transmission. The adult axonal translatome is unique, and its main role is likely to regulate synapse function. Developmentally regulated translatomes were subdivided according to the changes in translation between stages, and those that showed a coordinate change were found to share common upstream regulators, such as mTORC1, FMRP, and APC, as well as novel sequence elements that possibly regulate axonal mRNA translation. Additionally, we found that axonally translated mRNAs were frequently specific splice variants that carried axon-specific motifs. Together, our results show that extensive local mRNA translation occurs in the developing and mature mammalian CNS axons in vivo and provide strong evidence that highly regulated axonal mRNA translation might be at the heart of CNS development and the maintenance of synaptic function.

Previous studies using cultured neurons have revealed that some mRNAs are stored in a translationally repressed state (Buxbaum et al., 2014, Graber et al., 2013). Two independent and complementary approaches have been developed to ask which mRNAs are translated in the axon (Kim and Jung, 2015): metabolic labeling of newly synthesized proteins and isolation of ribosome-bound mRNAs. Proteomic approaches provide the ultimate readout of gene expression, as they can identify post-translationally modified protein products, but a critical limitation of proteomics is that the probe-tagged amino acid or its analogs label all cells, limiting its use to compartmentalized axon culture. An alternative strategy to identify newly synthesized proteins is to look at translated mRNAs (translatome), because these are the obligate precursor to the de novo proteome. A key advantage of this approach is that ribosomes can be isolated from a specific cell type by expressing a genetically encoded epitope-tagged ribosomal protein in just the cells of interest (TRAP; Heiman et al., 2008). Isolated ribosome-mRNA complexes either can be partially digested by RNase, and the fragments protected by ribosome binding can be sequenced (“ribosome profiling”; Ingolia et al., 2011), or the entire mRNA can be separated from the ribosome and directly sequenced. Only the latter method, which we use in this study, allows the discovery of novel isoforms outside the protein-coding region.

We compared translatomes of RGC axons and retinal cell bodies in the same animals. It should be noted that the retinal translatome includes the translatome of the short axons and dendrites of the intraretinal circuitry, as well as their cell bodies. Therefore, the number of mRNAs that are identified as selectively translated in the axon in this study may be an underestimation. Additionally, the expression of tagged ribosomes in non-RGC retinal neurons can potentially introduce bias into the axon/soma ratio. However, given the previous observations on retinal cell populations (Young, 1985), the presence of non-RGC mRNAs in the retinal sample cannot explain the axonal enrichment of mRNAs above the threshold (FPKM_axon_/FPKM_retina_ > 100), which we used for the axon-soma comparison. The strong enrichment of genes with axonal function in the axonal translatome compared to the retinal translatome suggests that these mRNAs were disproportionately represented in the axonal translatome, indicating that axonal translation is mRNA specific.

In this study, we show that the RGC axonal translatome changes in a developmental-stage-specific manner, in such a way that proteins playing a key role at specific periods are synthesized when needed. This result is in agreement with a recent study using the *Drosophila* visual system, which reported that neuronal differentiation associated with maturation of presynaptic terminals is regulated by coordinate control of mRNA translation (Zhang et al., 2016), although the subcellular location of mRNA translation was not addressed in that study. Whether signals that regulate mRNA-specific translation come from a cell-intrinsic timer or cell-extrinsic cues remains to be investigated, but our bioinformatic and experimental analyses suggest that this involves stage-dependent activation of RNA-binding proteins, including FMRP. Because FMRP is known to inhibit translation of proteins required for synapse formation and its loss of function leads to over-branching of CNS axons (references in Darnell and Richter, 2012), it is reasonable to assume that FMRP may be activated after CNS axons make appropriate synapses to limit the number of synapses that a single axon makes. In this sense, it is intriguing that defective translational machinery, which is expected to affect all cells in the organism, leads to enigmatically synapse-specific phenotypes, ranging from defective synaptic transmission to impaired cognitive function and memory (references in Buffington et al., 2014). Although the subcellular location of this pathogenesis is unknown, it will be interesting to test whether the axonal translatome of developing cortical neuronal axons in these mouse models of neurodevelopmental disorders is any different from normal mice.

The local mRNA translation in axons of mature neurons has been a subject of long-standing debate (Piper and Holt, 2004). Evidence indicates that ribosomes exist in mature CNS axons (Koenig et al., 2000, Kun et al., 2007, Walker et al., 2012) and that their number is dynamically regulated under normal and pathological conditions (Verheijen et al., 2014). However, what proteins are locally translated in the mature axons was unknown. Our comparative analysis of mature and developing retinal axonal translatomes suggests that local protein synthesis regulates synaptic transmission and axon maintenance. Because axonal translation has been implicated in axonal survival and degeneration (Jung et al., 2012), it will be important to find out whether pathological axon degeneration is preceded by defective axonal translation. The power of axon-TRAP is that it can be extended to other neurons whose cell bodies and axons are anatomically separated. One such example is cortical and spinal motor neurons, whose axonal degeneration leads to human diseases, such as amyotrophic lateral sclerosis (ALS). Recent evidence suggests that defective axonal mRNA transport and translation may be an underlying cause of ALS pathology (Alami et al., 2014, Murakami et al., 2015). Our new technical approach and datasets should provide a valuable resource for future studies.

## Experimental Procedures

### Histological Analysis

For immunohistochemistry, tissue sections (12 μm) were visualized using an anti-HA antibody (Abcam ab9110) and a secondary antibody conjugated to Alexa 488 (Life Technologies). For immuno-gold EM, tissues were fixed in 4% paraformaldehyde in 0.1 M HEPES (pH 7.4), and the HA-tagged ribosomes were visualized by the same anti-HA antibody and immunoglobulin G (IgG) conjugated with gold (10–15 nm). Mouse RGC axon culture and QIF were performed as previously described (Zivraj et al., 2010) using the following antibodies: anti-mTOR (phospho S2448) antibody (Abcam 109268), anti-FMRP antibody (Abcam 17722), and anti-RPS6 (phospho S235 + S236) antibody (Abcam 12864).

### Axon-TRAP

A homozygote RiboTag female mouse was mated with a Pax6-alpha-Cre male to produce Cre-positive and Cre-negative mice in a single litter. Three eyes or six SCs were homogenized, and post-mitochondrial fractions were collected. The mRNA-ribosome complexes were precipitated using the polyclonal HA antibody and Dynabeads Protein G (Life Technologies 10004D). For the in vitro ribosome run-off experiments, TRAP was performed after lysate was incubated with rabbit reticulocyte lysate (Promega), harringtonine (Sigma), and 4E1RCat (Sigma) at 37°C for 30 min. Ribosome-bound mRNAs were amplified by a method developed by Tang et al. (2009) with slight modification and sequenced using Illumina HiSeq2000 or NextSeq500. All experiments complied with protocols approved by the University of Cambridge and the Yonsei University College of Medicine Institutional Animal Care and Use Committees.

### Data Analysis

The sequence reads were mapped using TopHat 2 version 2.0.12, and FPKM values were estimated using Cufflinks. Read counts for each gene were determined using HTSeq version 0.6.1p1. For the identification of translated mRNAs in RGC axons, we applied differential gene expression analysis on read count using NOISeq. De novo motif analysis was performed using HOMER version 3.0 with custom FASTA files.

A detailed description of all experimental procedures is provided in the Supplemental Information.

## Author Contributions

H.J. and C.E.H. conceived and supervised the project. H.J. performed histological experiments. H.J. and T.S. performed biochemical and molecular biological experiments. T.S. performed bioinformatic analyses. J.J. performed Cre specificity experiments, J.O. performed motif imaging and QIF, and B.T.-B. and J.Q.L. performed FRAP and QIF. P.S.A. provided RiboTag mice and the original TRAP protocol. H.J., T.S., and C.E.H. wrote the manuscript.

## Accession Numbers

The accession number for the RNA-seq data reported in this paper is GEO: GSE79352.

## Supplemental Information

Document S1. Supplemental Experimental Procedures

Table S1. Axonally Translating mRNAs, Related to Figure 3

Table S2. Neuron-Related GO Terms, Related to Figure 4

Table S3. GO Term and Motif Analysis, Related to Figure 5

Document S2. Article plus Supplemental Information
