# Supplementary material for: Dynamic Axonal Translation in Developing and Mature Visual Circuits
Source: Cell. 2016 Jun 30;166(1):181–92. doi: 10.1016/j.cell.2016.05.029 (PMC4930487; doi:10.1016/j.cell.2016.05.029)
Supplement: Document S1. Supplemental Experimental Procedures [file mmc1.pdf]

Cell, Volume *166*

Supplemental Information

Dynamic Axonal Translation in Developing and Mature Visual Circuits

Toshiaki Shigeoka, Hosung Jung, Jane Jung, Benita Turner-Bridger, Jiyeon Ohk, Julie Qiaojin Lin, Paul S. Amieux, and Christine E. Holt

SUPPLEMENTAL INFORMATION

The dynamic translatome of retinal ganglion cell axons during assembly and maintenance of the mouse visual system

Toshiaki Shigeoka[1,4], Hosung Jung[2,4, 5], Jane Jung[2], Benita Turner-Bridger[1], Jiyeon Ohk[2], Julie Qiaojin Lin[1], Paul S. Amieux[3], Christine E. Holt[1,5]

[1]Department of Physiology, Development and Neuroscience, University of Cambridge, Downing Street, Cambridge, CB2 3DY, United Kingdom.

[2]Department of Anatomy, Brain Research Institute, and Brain Korea 21 PLUS Project for Medical Science, Yonsei University College of Medicine, Seoul, 03722, Republic of Korea.

[3]Bastyr University Research Institute, Bastyr University, Kenmore, WA 98028, USA.

[4]Co-first author

[5]Co-senior author

*Correspondence should be addressed to H.J. (hosungjung@yonsei.ac.kr) or C.E.H. (ceh33@cam.ac.uk)

EXTENDED EXPERIMENTAL PROCEDURES

Animals

RiboTag and Pax6-alpha-Cre mice were kind gifts from Dr Paul Amieux (University of Washington) and Dr Peter Gruss (Max Planck Institute), respectively. A homozygote RiboTag female mouse was mated with a Pax6-alpha-Cre male, to produce Cre-positive and Cre-negative mice in a single litter. Cre-negative embryos or pups were used as negative controls for TRAP. Rosa26-StopLox-LacZ and Rosa26-StopLox-TauLacZ were kindly provided by Dr Jin-Woong Bok (Yonsei University) and Dr Soochul Park (Sookmyung Woman's University), respectively. All procedures were conducted under license in accordance with UK Home office guidelines and under the Guidelines for the Care and Use of Laboratory Animals of Yonsei University College of Medicine.

Monitoring of Cre-recombinase activity in Pax6-alpha-Cre mice

As even slightly leaky expression of the HA-tagged ribosome in any SC-resident cells would lead to misidentification of axonally translating mRNAs, we needed to confirm that no resident cells in the SC express Cre. We took two independent approaches. In the first, we used two Cre-responsive reporter mice: one that labels the cell bodies of Cre-expressing cells and their progeny (Rosa26-StopLox-LacZ) and the other that labels the axons of these cells (Rosa26-StopLox-TauLacZ). In accordance with the previous reports (Marquardt et al., 2001), LacZ-positive cell bodies were only observed in the neural retina but not in the SC (Fig. S1). Using the StopLox-TauLacZ reporter mouse to visualize retinal axons, we could see the SC is richly innervated by the axons originating from their Cre-positive cell bodies in the retina (Fig. S1). In the second approach, we crossed this mouse with RiboTag (Fig. 1C), and asked whether we could detect any trace of the recombined *RiboTag* allele (*HA-rpL22)* in the SC. If the SC contains any resident cells that have expressed Cre but escaped our

histological analysis, the nuclear DNA extracted from this tissue must contain the *HA-rpL22* allele, which we can detect using PCR-based assays (Fig. 1D). We detected no such signal in the SC dissected for TRAP (Figs. 1D and S1, red box). Therefore, both histological and molecular biological assays confirm that the only source of HA-tagged ribosomes in the SC is the RGC axons. Genotyping was performed by polymerase chain reaction (PCR) using the following primer pairs: for the Cre transgene, forward 5'- GCATTACCGGTCGATGCA ACGAGTG-3', and reverse 5'- GAACGCTAGAGCCTGTTTTGCACGTTC-3'; for the RiboTag allele, forward 5'-GGGAGGCTTGCTGGATATG-3, and reverse 5'- TTTCCAGACACAG-GCTAAGTACAC-3'; for detection of the recombined RiboTag allele (HA-rpL22), forward 5'-TTCTCTAGAAAGTATAGGAACTT-3', and reverse 5'-ACATCGTATGGG-TATAGATCC-3'. Cre-negative embryos or pups were used as negative controls for TRAP.

Quantitative reverse transcription-polymerase chain reaction (qRT-PCR)

cDNA was synthesized using an oligo-dT primer and SuperScript III reverse transcriptase (Invitrogen). The qPCR was performed using QuantiTect SYBR Green PCR Kit (Qiagen) on Light Cycler LC480 II (Roche). The levels were normalized by the total RNA amounts. The following primer pairs were used: for Glud1, forward 5'-GGGAGGTCATCGAAGGCTAC-3', and reverse 5'-AGCCAGTGCTTTTACTTCAT-CC-3'; for Mapt, forward 5'-TTCTGTCCTCGCCTTCT-GTC-3', and reverse 5'-CCTTCTTGGTCTTGGAGCAG-3'; for Rps5, forward 5'-TCAAGCTCTTTGGGAAAT-GG-3', and reverse 5'-GGGCAGGTACTTGGCATACT-3'; for Tsc2, forward 5'-TAGGGCTCCTGGTCATCCTT-3', and reverse 5'-GTGCT-TGTAATGGAGCTGGA-3'; for Cfl1, forward 5'-TCTGTCTCCCTTTCGTTTCC-3', and reverse 5'-GCCTTCTTGCGTTTCTTCAC - 3', for Aldoa, forward 5'-TTAGTCCTTTCGCCTA-CCCA-3', and reverse 5'-AGCTCCTTCTTCTGCTCCG-3'; for Atp5b, forward 5'-CACAATGCAGGAAAGGATCA-3', and reverse 5'-GGGTCAGTCAGG-TCATCAGC-

3'; for Basp, forward 5'-ACAAAGACAAGAAGGCCGAA-3', and reverse 5'-CTCTCCTTGACCTCGGTGG-3'; and for Cend1, forward 5'-CCTGAGCACT-CCTCGGTATC-3', and reverse 5'-AGACCACAGTGGCTCAGGAC-3'.

Histological analysis

Mouse embryos were fixed by immersion in 4% paraformaldehyde in PBS. Adult mice were transcardially perfused with the same fixative, and entire eyes and brains were dissected out and post-fixed. For X-gal staining, brains and retinae were dissected out, washed in ice-cold PBS and fixed for 1 hour in 1% formaldehyde, 0.2% glutaraldehyde, 2mM $MgCl_2$, 5mM EGTA, 0.02% NP-40 in PBS [pH7.5]) at 4°C on a shaker. The dissected tissues were rinsed three times for 20 min each in PBS and stained with X-gal staining solution (1mg/ml X-gal diluted in DMF, 0.01% Sodium deoxycholate, 0.02% NP-40, 5 mM potassium ferricyanide, 2 mM $MgCl_2$ in PBS) between 3 to 48 hours at 37°C in the dark. After washing with PBS, the tissues were post-fixed in 4% paraformaldehyde in PBS for 30 min at 4°C. For immunohistochemistry, tissues were saturated in 30% sucrose, embedded in OCT, frozen on dry ice, sectioned at 12 μm by using a cryostat (Leica CM3050S), and then visualized using an anti-HA antibody (Abcam ab9110) and a secondary antibody conjugated to Alexa Fluor 488 (Life technologies). For immuno-gold electron microscopy, tissues were fixed in 4% paraformaldehyde in 0.1M HEPES (pH 7.4), and the HA-tagged ribosomes were visualized by the same anti-HA antibody and IgG conjugated with gold (10nm-15nm) at the Cambridge Advanced Imaging Centre (University of Cambridge, UK). Specific labeling was evident as ultrastructurally identifiable ribosomes were labeled by multiple gold particles (Fig. 1F, purple arrows). We did, however, occasionally observe scattered gold particles in the Cre-negative tissue (Fig. 1G, white arrow), but these were never clustered as seen in the Cre-positive group. Therefore, we considered only two or more gold particles in close

proximity (within 50 nm between particles) as specific labeling of HA-positive ribosomes.

Axon-Translating Ribosome Affinity Purification (TRAP)

Tissue samples were dissected and snap-frozen in liquid nitrogen and genotyped. Whole eyes were used as the cell body group and the superior colliculus, where retinal axons terminate, were used as the axon terminal group. Tissues from cre-positive and negative were pooled for TRAP (three eyes and superior colliculi for one group). Tissues were homogenized in lysis buffer (20mM HEPES-KOH, 5mM $MgCl_2$, 150mM KCl, 1mM DTT, SUPERase In, and Complete EDTA-free Protease Inhibitor Cocktail) in the presence of cycloheximide (to stop translational elongation and to lock translating ribosomes on the mRNA) and rapamycin (to prevent new translational initiation during immunoprecipitation), and post-mitochondrial fractions were collected. We optimized the TRAP protocol before performing axon-TRAP. First, we found the polyclonal HA antibody (9110, Abcam) is superior to the one used in the original RiboTag study (HA11, Covance) (Sanz et al., 2009). Although the two antibodies were similarly effective in precipitating HA-rpL22, the polyclonal antibody (Abcam ab9110) co-purified much more 80S ribosomes (Fig. 2A). We estimated that approximately 40% of HA-tagged translating ribosomes could be purified this way, as the amount of TRAPed 80S ribosomes (i.e. co-immunoprecipitated rpS3a, a 40S ribosomal protein) was approximately 10% of the input (i.e. rpS3a in total input) (Fig. 2A). This estimation is based on the findings that (1) roughly 50% of retinal cells express HA-tagged rpL22 (i.e. 50% cells do not express tagged ribosomes), and that (2) these cells express rpL22 from one wildtype allele and one recombined HA-rpL22 allele (*i.e*. 50% of rpL22 is labeled with HA in Cre-positive cells) ([total 80S] = [HA-80S] x (1/50%) x (1/50%) = [TRAPed HA-80S] x (1/10%). Therefore, [TRAPed HA-80S] = (4/10) x [HA-80S]) (Fig. 2A). We found that the indirect immunoprecipitation protocol, which allows the antibody to bind the antigen before the purification of the

antigen-antibody complexes, was more specific than the direct protocol, which utilizes the antibody pre-conjugated to Protein G-magnetic beads. We think that nonspecific binding of mRNAs to Protein G-magnetic beads was reduced in the indirect protocol, as the tissue lysate was pre-cleared with unconjugated Protein G-magnetic beads. Pre-cleared ribosome-mRNA complexes were immunoprecipitated by an anti-HA antibody and Dynabeads Protein G (Life Technologies 10004D). Total RNA was extracted from the ribosome-mRNA complexes using an RNeasy mini kit (Qiagen) followed by in-column DNase treatment to remove genomic DNA contamination. The RNA samples were examined for quantity and quality using the Agilent Bioanalyzer 2100 (Agilent Technologies).

Amplification of cDNAs

The amount of axon-TRAPed mRNA was minute and had to be amplified (Fig. S2A). We used a method developed by Tang and colleagues for single cell transcriptomics (Tang et al., 2009) with slight modification (Fig. S2A). First, TRAPed RNAs were treated with DNase I to eliminate genomic DNA contamination and then mRNAs were reverse-transcribed with oligo(dT) primer with a linker. After second strand synthesis with another linker, the double-strand cDNAs were amplified by ten rounds of PCR and visualized by agarose gel electrophoresis (Fig. 2B). The specificity of TRAP was evident because it was dependent on Cre. As expected, the amount of TRAPed mRNAs was lower in the SC (mRNAs from RGC axons) than in the eye (mRNAs from the cell body and proximal neurites), and therefore was further amplified by five additional rounds of PCR. Although axon-TRAP was clearly dependent on Cre and therefore specific, increasing PCR cycles led to an increased background (i.e. amplified cDNAs from the Cre-negative SC). We reasoned that the amplified DNAs in the Cre-negative SC would reflect the relative abundance of mRNAs in the SC. We sequenced these mRNAs and used the data as a negative control for bioinformatics analysis of axon-TRAPed mRNAs (Fig. 2B). Amplified

cDNAs were subjected to paired-end 100 or 90 bp sequencing on the Illumina HiSeq2000.

For the *in vitro* ribosome run-off experiment, eyes or superior colliculi were homogenized by the 400 µl lysis buffer without cycloheximide, and then 200 µl of rabbit reticulocyte lysate (Promega) and 8 µl of Harringtonine (100 ug /ml) and 4 µl of 4E1RCat (5 mM) were added to the 188 µl lysate, followed by 37 °C incubation for 30 min. To stop the *in vitro* translation elongation, 800 µl of ice-cold lysis buffer with cycloheximide was added, followed by immunoprecipitation with the anti-HA antibody. The purified RNAs were subjected to RNA sequencing on the Illumina NextSeq 500. To reflect the difference in amount of amplified cDNAs between the samples with and without run-off translation on the sequencing depth, we first measured the amount of cDNA by both Tapestation DNA and by Qubit. We then adjusted the ratio between the amounts of input libraries for multiplex sequencing using the ratio of cDNA amount between the two samples. All RNA-seq data are deposited in Gene Expression Omnibus (GEO) datasets under accession number GSE79352.

Mapping of sequence reads and normalization of read counts

The sequence reads were mapped to the mouse genome (mm10) using TopHat 2 version 2.0.12 (Kim et al., 2013) with default settings, except for the "--read- realign-edit-dist 0" option. This option was chosen to reduce mapping to pseudogenes. Although we detected a significant number of sequencing reads of the primer dimers in several samples, which are probably caused by the matching problem between our primers and Illumina primers, because there is no biological reason that these dimers bias the relative abundance of sequence reads of endogenous mRNAs, we analyzed all sequence reads that can be mapped to the mouse genome sequence by TopHat 2. Transcript assembly and estimation of FPKM (Fragments Per Kilobase of

transcript per Million fragments sequenced) values were performed using Cufflinks version 2.2.1 (Trapnell et al., 2010). Read counts for each gene were determined using HTSeq version 0.6.1p1 (http://www- huber.embl.de/users/anders/HTSeq/). For the analysis of run-off samples, we used the read counts for all DEGs (adult) detected either in the run-off positive or negative sample.

Differential gene expression analysis and Gene Ontology based enrichment analysis

For the identification of translated mRNAs in RGC axons, we applied the differential gene expression analysis on read count data between two biological replicates of Cre-positive and Cre-negative samples using NOISeq (Tarazona et al., 2011) in default conditions with probability threshold 0.7. Although this approach has a risk of filtering out actively translating mRNAs if the same mRNAs exist in high abundance in SC-resident cells and are proportionately represented in the negative control, we thought that it would be appropriate to take a conservative approach when analyzing mRNAs identified by highly sensitive RNA-seq from samples with potentially low signal-to-noise ratio. DEG analysis identified a subset of Cre-dependent mRNAs (Fig. 2D, right panel, red dots) (Table S1). In some cases, genes that passed our filtering criteria and were identified as DEGs in one stage failed the same test in other stages (Fig. 2D, orange), because their abundance in the negative control varied depending on the stage. Most of these mRNAs, however, were found in the axon-TRAPed mRNAs at all stages tested, suggesting that these genes became false negatives in other stages. Therefore, when we analyzed the developmental change in translation levels, we used the FPKM (fragments per kilobase of transcript per million mapped reads) values of all four stages for genes that have passed the DEG test in at least one stage (Union of DEGs) (Fig. 2D, orange and peach).

To perform a GO-based analysis for neuronal functions (Figs. 4A and S4A), we selected 455 neuron-related GO terms (Table S2) using the following criteria: all offspring GO terms (249 terms) of ‘Neuron development (GO:0048666)’, which contain GO terms related to neuron/axon development, and all offspring GO terms (206 terms) of "Synaptic transmission (GO:0007268)", which contain GO terms related to neurotransmission. The offspring GO terms were identified using the GOBPOFFSPRING function of GO.db, an R package (Table S2). The enrichment analysis for gene ontology was carried out with DAVID and topGO version 2.18. (Alexa et al., 2006) on R version 3.1.2. The result for all analyzed GO terms (biological process) is represented in Table S3. For Kyoto Encyclopedia of Genes and Genomes (KEGG) analysis, we used all 2576 axonally translated genes and calculated p values and fold enrichments using DAVID (Fig. 4A, lower panel). The numbers of GO terms associated with retinal and axonal translatome were analysed using ClueGO (Bindea et al., 2009). Statistically significant enrichment is annotated by a solid box outline.

Ingenuity Pathway Analysis

Ingenuity Pathway Analysis (Qiagen) for canonical pathways (Fig. 5A) was performed according to the manufacturer’s instructions. For quantitative analysis of stage-dependent axonal translation of each mRNA, we calculated the ratio in read counts of each mRNA from one stage to the next. Then we performed Ingenuity Pathway Analysis (IPA) for upstream regulators, which mainly utilizes published results of gene knockdown or knockout studies. For example, if the protein product of a gene decreases when a specific translational regulator is knocked out, the gene would be described as “positively regulated” by the translational regulator (and vice versa) (Fig. 5B).

Analysis of target mRNAs in the mTOR pathway and RNA binding proteins

We analyzed the known binding targets of RBPs using the results of previous studies on FMRP, TDP-43, FUS and APC. In addition, we carried out the same analysis on the targets of mTORC1 to confirm the result of IPA analysis. If any of these molecules regulate stage-specific translation in the axon, their target mRNAs would show coordinate changes in translation. We used the gene sets that were identified in previous studies (Colombrita et al., 2012; Darnell et al., 2011; Preitner et al., 2014; Thoreen et al., 2012). We analyzed mTOR targets that are described in the previous report (Thoreen et al., 2012) with a threshold of log2 (Torin1/Vehicle) < -1, FMRP targets (Darnell et al., 2011) with a threshold of rank (based upon chi-square score) < 100 and all of the APC targets (Preitner et al., 2014), TDP-43 targets and FUS targets identified(Colombrita et al., 2012). In the FPKM ratio analysis for consecutive developmental stages, we used all genes that were detected in RNA-seq in order to avoid any bias caused by differential gene expression analysis. Principle component analysis (PCA) was performed on normalized read counts of all samples to compare gene expression in 12 different conditions by using the “prcomp” function in the R Software package (version 2.13.0). Data were plotted using the first two PCs which explained up to 73.2% of the total variance: 66.1% explained by PC1; and 7.1% by PC2.

Analysis of mRNA isoforms

Alternative isoforms were analyzed on mapped reads from the P0.5 sample using MISO. Before this analysis, the mean and the standard deviation of the insert length and the total number of mapped read pairs were computed using the “pe_utils” utilities (Retina: mean=177.8, sdev=9.7, dispersion=0.7, num_pairs=3262858, Axon: mean=178.3, sdev=13.5, dispersion=1.0, num_pairs=2535309, Cre_negative axon: mean=179.6, sdev=9.2, dispersion=0.7, num_pairs=3235484). We used Mouse genome (mm10) alternative events version 1.0 (Wang et al., 2008) to perform “exon-centric” analyses. We counted and analyzed only the events in which both splicing

variants are detected both in axon and in cell body (the events of “0 < Ψ <1" both in axon and in cell body). For the discovery of novel splice variants, we performed reference-independent transcript reconstruction using Cufflinks version 2.2.1 (Trapnell et al., 2010) and compared the sequences of reconstructed transcripts with the UCSC Genes transcript annotations by using the Cuffcompare program from the Cufflinks package. For the identification of circular RNAs, we first extracted the fusion transcripts from the unmapped sequence reads by using TopHat-fusion (Kim and Salzberg, 2011) (TopHat 2.0.12, parameters:--fusion-search --keep-fasta-order --bowtie1 --no-coverage-search), and then identified junction reads from back spliced exons using CIRCexplorer-1.1.1(Zhang et al., 2014). Mapped sequence reads are visualized using the Integrative Genomics Viewer (IGV) version 2.3.46 (Robinson et al., 2011).

Identification of regulatory motifs in RNA

De novo motif analysis was performed using HOMER version 3.0 (Heinz et al., 2010) with custom FASTA files. For the finding of motifs in UTRs, we used the UTR sequences of genes whose levels were higher in the axon than in the retina (axon / retina > 100, Fig. 3A) in P0.5 translatomes. For finding of motifs in axon-enriched alternative exons, detection of differential exon usage was performed using DEXseq version 1.10.8 (Anders et al., 2012) on R version 3.1.0, and exons were selected with a Benjamini–Hochberg adjusted p-value cut-off of < 0.1 (Benjamini and Hochberg, 1995). To validate these motifs, we extracted the genes that contain these motifs allowing 0-1 mismatch and then compared the translation levels between axon and retina. Sequences of UTRs were retrieved from Ensemble BioMart (Kinsella et al., 2011). Motif containing genes were identified using the Biostrings package on R version 3.1.2.

Immunofluorescence

Mouse RGC axons were cultured as previously described (Zivraj et al., 2010). The cultures were fixed in 2% (vol/vol) paraformaldehyde/7.5% (wt/vol) sucrose, permeabilized with 0.1% Saponin (Sigma) and blocked in 5% goat serum, then labeled with primary antibodies overnight and Alexa Fluor 488 secondary antibodies (1:1000, Life Technologies), and mounted in FluorSave (Calbiochem). Randomly selected isolated axons were imaged with a Plan Apo 60X oil objective on a Nikon Eclipse TE2000-U inverted fluorescent microscope with a Hamamatsu ORCA-ER CCD camera. Exposure time was kept constant and below greyscale pixel saturation. For quantitation of fluorescence intensity, the outline of the axon segments with similar lengths was traced on the phase image using Volocity software (PerkinElmer), and then superimposed on the fluorescent image. The software calculated the fluorescent intensity within the axon segment, giving a measurement of pixel intensity per unit area. The axon outline was then placed in an adjacent area clear of cellular material to record the background fluorescent intensity. This reading was subtracted from the axon reading, yielding the background-corrected intensity. Each reading was normalized to the mean fluorescent intensity of E17.5 axons in the same group. The fluorescent intensities of between 50 to 100 axon segments per sample group were collected. The following antibodies were used: anti-mTOR (phospho S2448) antibody (Abcam 109268), anti-FMRP antibody (Abcam 17722), and anti-RPS6 (phospho S235 + S236) antibody (Abcam 12864).

Fluorescence recovery after photobleaching (FRAP)

Motif sequences were cloned into a plasmid containing the coding sequence of membrane-targeted, destabilized EGFP (myr-d2EGFP) (Aakalu et al., 2001) between NotI-Xhoi sites (for 3'-UTR and alternative exon) or NheI-BamHI sites (for 5'-UTR). Motif sequences are in Table S3. The reporter plasmids were expressed in the retina by targeted electroporation into *Xenopus laevis* embryos, and fluorescence was imaged in cultured retinal ganglion cell axons as previously described (Leung et

al., 2006), except that 1% fetal bovine serum was added to the medium. In experiments to confirm translation-dependency of FRAP, the translation inhibitor anisomycin (40 µM) was added 30 min prior to imaging. FRAP experiments were performed on an Olympus IX81 inverted microscope equipped with a PerkinElmer Spinning Disk UltraVIEW VoX and a 60x (NA, 1.30) Olympus silicone oil immersion objective. Images were acquired with an ORCA-Flash4.0 V2 CMOS camera (Hamamatsu) using Volocity software (PerkinElmer).

Photobleaching was performed using an UltraVIEW PhotoKinesis device (PerkinElmer). Regions of interest were manually defined so that growth cones and ≥20µm of the axon shaft were bleached (thus reducing likelihood of fluorescence recovery resulting from myr-d2EGFP diffusion from unbleached areas of the axon shaft). Photobleaching was performed at 50-85% laser power (488 nm laser line) with 20–30 bleach cycles. Time-lapse images were captured at 1 min intervals using a 488 nm laser line at 25% laser power for myr-d2EGFP and a 561 nm laser line (31 % laser power) for visualization of axons using a membrane-targeted mCherry-CAAX reporter, in addition to phase contrast. Exposure time for the 488 channel was adjusted to avoid pixel saturation and was typically between 50-200ms. Quantification of fluorescence intensity was performed using Volocity software (PerkinElmer). At each time point, the outline of the growth cone (ROI) was traced using the mCherry-CAAX reporter (561 channel) and phase contrast images. Mean gray values from the 488 channel were subsequently calculated as mean pixel intensity per unit area within the ROI. Subtracting the mean background pixel intensity per unit area from an equivalently sized ROI immediately adjacent to the growth cone normalised intensity values in the growth cone. Unhealthy axons exhibiting signs of photo-toxicity after FRAP (characterised by blebbing, growth cone collapse and/or retraction) were excluded from analysis. In addition, we only quantified growth cones of axons extending more than 100 µm from the eye explant

to reduce effects of somal diffusion. Relative fluorescent recovery (R) at each time point was calculated by the formula: $R_x = (I_x – I_{post}) / (I_{pre} – I_{post})$. Where, I_x = normalised fluorescent intensity of the growth cone ROI at time point 'x', I_{pre} = normalised fluorescent intensity before photobleaching and I_{post} = normalised fluorescent intensity immediately after photobleaching (t=0 mins).

Data were analysed using PRISM software (Graphpad). Significance was tested using a two-way ANOVA.

REFERENCES

Aakalu, G., Smith, W.B., Nguyen, N., Jiang, C., and Schuman, E.M. (2001). Dynamic visualization of local protein synthesis in hippocampal neurons. Neuron *30*, 489-502.

Anders, S., Reyes, A., and Huber, W. (2012). Detecting differential usage of exons from RNA-seq data. Genome research *22*, 2008-2017.

Benjamini, Y., and Hochberg, Y. (1995). Controlling the False Discovery Rate - a Practical and Powerful Approach to Multiple Testing. J Roy Stat Soc B Met *57*, 289-300.

Colombrita, C., Onesto, E., Megiorni, F., Pizzuti, A., Baralle, F.E., Buratti, E., Silani, V., and Ratti, A. (2012). TDP-43 and FUS RNA-binding proteins bind distinct sets of cytoplasmic messenger RNAs and differently regulate their post-transcriptional fate in motoneuron-like cells. J Biol Chem *287*, 15635-15647.

Darnell, J.C., Van Driesche, S.J., Zhang, C., Hung, K.Y., Mele, A., Fraser, C.E., Stone, E.F., Chen, C., Fak, J.J., Chi, S.W.*, et al.* (2011). FMRP stalls ribosomal translocation on mRNAs linked to synaptic function and autism. Cell *146*, 247-261.

Heinz, S., Benner, C., Spann, N., Bertolino, E., Lin, Y.C., Laslo, P., Cheng, J.X., Murre, C., Singh, H., and Glass, C.K. (2010). Simple combinations of lineage-determining transcription factors prime cis-regulatory elements required for macrophage and B cell identities. Molecular cell *38*, 576-589.

Kim, D., Pertea, G., Trapnell, C., Pimentel, H., Kelley, R., and Salzberg, S.L. (2013). TopHat2: accurate alignment of transcriptomes in the presence of insertions, deletions and gene fusions. Genome biology *14*, R36.

Kim, D., and Salzberg, S.L. (2011). TopHat-Fusion: an algorithm for discovery of novel fusion transcripts. Genome biology *12*, R72.

Kinsella, R.J., Kahari, A., Haider, S., Zamora, J., Proctor, G., Spudich, G., Almeida-King, J., Staines, D., Derwent, P., Kerhornou, A.*, et al.* (2011). Ensembl BioMarts: a hub for data retrieval across taxonomic space. Database : the journal of biological databases and curation *2011*, bar030.

Leung, K.M., van Horck, F.P., Lin, A.C., Allison, R., Standart, N., and Holt, C.E. (2006). Asymmetrical beta-actin mRNA translation in growth cones mediates attractive turning to netrin-1. Nat Neurosci *9*, 1247-1256.

Marquardt, T., Ashery-Padan, R., Andrejewski, N., Scardigli, R., Guillemot, F., and Gruss, P. (2001). Pax6 is required for the multipotent state of retinal progenitor cells. Cell *105*, 43-55.

Preitner, N., Quan, J., Nowakowski, D.W., Hancock, M.L., Shi, J., Tcherkezian, J., Young-Pearse, T.L., and Flanagan, J.G. (2014). APC is an RNA-binding protein, and its interactome provides a link to neural development and microtubule assembly. Cell *158*, 368-382.

Robinson, J.T., Thorvaldsdottir, H., Winckler, W., Guttman, M., Lander, E.S., Getz, G., and Mesirov, J.P. (2011). Integrative genomics viewer. Nature biotechnology *29*, 24-26.

Sanz, E., Yang, L., Su, T., Morris, D.R., McKnight, G.S., and Amieux, P.S. (2009). Cell-type-specific isolation of ribosome-associated mRNA from complex tissues. Proc Natl Acad Sci U S A *106*, 13939-13944.

Tang, F., Barbacioru, C., Wang, Y., Nordman, E., Lee, C., Xu, N., Wang, X., Bodeau, J., Tuch, B.B., Siddiqui, A., *et al.* (2009). mRNA-Seq whole-transcriptome analysis of a single cell. Nat Methods *6*, 377-382.

Tarazona, S., Garcia-Alcalde, F., Dopazo, J., Ferrer, A., and Conesa, A. (2011). Differential expression in RNA-seq: a matter of depth. Genome research *21*, 2213-2223.

Thoreen, C.C., Chantranupong, L., Keys, H.R., Wang, T., Gray, N.S., and Sabatini, D.M. (2012). A unifying model for mTORC1-mediated regulation of mRNA translation. Nature *485*, 109-113.

Trapnell, C., Williams, B.A., Pertea, G., Mortazavi, A., Kwan, G., van Baren, M.J., Salzberg, S.L., Wold, B.J., and Pachter, L. (2010). Transcript assembly and quantification by RNA-Seq reveals unannotated transcripts and isoform switching during cell differentiation. Nature biotechnology *28*, 511-515.

Wang, E.T., Sandberg, R., Luo, S., Khrebtukova, I., Zhang, L., Mayr, C., Kingsmore, S.F., Schroth, G.P., and Burge, C.B. (2008). Alternative isoform regulation in human tissue transcriptomes. Nature *456*, 470-476.

Zhang, X.O., Wang, H.B., Zhang, Y., Lu, X., Chen, L.L., and Yang, L. (2014). Complementary sequence-mediated exon circularization. Cell *159*, 134-147.

Zivraj, K.H., Tung, Y.C., Piper, M., Gumy, L., Fawcett, J.W., Yeo, G.S., and Holt, C.E. (2010). Subcellular profiling reveals distinct and developmentally regulated repertoire of growth cone mRNAs. J Neurosci *30*, 15464-15478.
